# Adaptation to zinc restriction in *Streptococcus agalactiae*: role of the ribosomal protein and zinc-importers regulated by AdcR

**DOI:** 10.1128/msphere.00614-24

**Published:** 2024-10-31

**Authors:** M. Melet, S. Blanchet, P. Barbarin, E. A. Maunders, S. L. Neville, V. Rong, L. Mereghetti, C. A. McDevitt, A. Hiron

**Affiliations:** 1 ISP, Université de Tours, Tours, France; 2 Department of Microbiology and Immunology, The Peter Doherty Institute for Infection and Immunity, University of Melbourne, Melbourne, Australia; 3 CHRU de Tours, Service de Bactériologie-Virologie Hygiène, Tours, France; The University of Arizona, Tucson, Arizona, USA

**Keywords:** zinc homeostasis, *Streptococcus agalactiae*, gene regulation, duplicated ribosomal proteins, metal ABC-transporter

## Abstract

Zinc (Zn) is an essential cofactor for numerous bacterial proteins and altering Zn availability is an important component of host innate immunity. During infection, adaptation to both Zn deprivation and excess is critical for pathogenic bacteria development. To understand the adaptive responses to Zn availability of *Streptococcus agalactiae*, a pathogen causing invasive infections of neonates, global transcriptional profiling was conducted. Results highlight that in response to Zn limitation, genes belonging to the AdcR regulon, the master regulator of Zn homeostasis in streptococci, were overexpressed. Through a combination of *in silico* analysis and experimental validation, new AdcR-regulated targets were identified. Among them, we identified a duplicated ribosomal protein, RpsNb, and an ABC transporter, and examined the role of these genes in bacterial growth under Zn-restricted conditions. Our results indicated that, during Zn restriction, both the RpsNb protein and a potential secondary Zn transporter are important for *S. agalactiae* adaptation to Zn deficiency.

**IMPORTANCE:**

*Streptococcus agalactiae* is a bacterial human pathobiont causing invasive diseases in neonates. Upon infection, *S. agalactiae* is presented with Zn limitation and excess but the genetic systems that allow bacterial adaptation to these conditions remain largely undefined. A comprehensive analysis of *S. agalactiae* global transcriptional response to Zn availability shows that this pathogen manages Zn limitation mainly through upregulation of the AdcR regulon. We demonstrate that several AdcR-regulated genes are important for bacterial growth during Zn deficiency, including human biological fluids. Taken together, these findings reveal new mechanisms of *S. agalactiae* adaptation under conditions of metal deprivation.

## INTRODUCTION

*Streptococcus agalactiae*, commonly known as group B *Streptococcus* or GBS, is a Gram-positive, beta-hemolytic, opportunistic pathogen (1–3). In humans, *S. agalactiae* asymptomatically colonizes the gastrointestinal and urogenital tracts, with a detected prevalence of approximately 18% in pregnant women (2, 4, 5). During delivery, there is a risk of mother-to-newborn transmission that can lead to invasive infections resulting in approximately 150,000 infant deaths globally each year, and more than 500,000 preterm births that frequently develop substantial long-term disabilities (6).

First-row transition metals, such as manganese, iron, and zinc (Zn), are essential for life due to their crucial roles as structural and/or catalytic cofactors in metalloproteins (7). Maintenance of intracellular metal ion concentrations is vital for bacterial viability and during infection, pathogenic bacteria must acquire essential nutrients from host tissues (8). Zn is the second most abundant transition metal in humans, although it is predominantly sequestered within cells (9). During infection, the vertebrate host immune system can reduce the relative bioavailability of Zn using metal-chelating proteins, impeding the propagation of some invading microbes (10, 11). This process is collectively referred to as nutritional immunity and involves host proteins such as calprotectin (8, 12).

To overcome the host-imposed restriction of Zn bioavailability, pathogenic bacteria employ various strategies (13). Frequently, this involves the expression of high-affinity uptake systems, such as the Adc transporter of *Streptococcus pneumoniae*, or secreted Zn-binding metallophores, such as the Cnt system of *Staphylococcus aureus* (14, 15). In *S. agalactiae,* Zn acquisition is primarily mediated by the Adc/Lmb system which is vital for bacterial growth in Zn-restricted environments and contributes to virulence *in vivo* (16–18).

Concomitant with the upregulation of metal acquisition system genes, bacteria can also access cytoplasmic metal stores. In *B. subtilis*, Zn serves as a cofactor for bacterial proteins ribosomal proteins L36, L33, L31 (50S subunit), and S14 (30S subunit) (19). Ribosomal proteins represent a major portion of the bacterial cellular Zn quotient, with each ribosome containing ~6–8 molecules of Zn and each cell producing more than 30,000 ribosome complexes during the exponential phase of growth (20). Genes encoding these Zn-containing ribosomal proteins (Zn+ form) are duplicated in *B. subtilis* and that their paralogues lack a CXXC Zn-binding motif (Zn− form) (21). In the case of the L31 protein, the Zn− form is overexpressed during Zn deficiency and can substitute for the Zn+ form within a pre-existing ribosome. The displaced Zn+ form is then rapidly degraded, releasing a significant amount of Zn (up to 20% of its intracellular Zn) for other enzymes in case of severe Zn restriction (8, 22).

The essentiality of Zn necessitates that the bacterial response to limitation is tightly regulated. In the proteobacteria and Gram-positive bacteria of the *Bacillus* species, Zn limitation is sensed by Zur, a metalloregulatory protein of the Fur family, while in streptococci and lactococci*,* this function is performed by AdcR/ZitR, a regulator of the MarR family (21, 23). In the presence of Zn, AdcR adopts a Zn^di^-AdcR conformation and inhibits target genes by binding to a palindromic sequence named the AdcR box (TTAACNNGTTAA) (21, 24–26). In Zn-limited environments, AdcR dissociates from the AdcR box, allowing the expression of target genes (18, 27, 28). The AdcR regulons of *S. pneumoniae* and *Streptococcus pyogenes* comprise 15 and 8 genes, respectively, mainly composed of genes encoding the Adc permease or accessory factors (26, 29). In *S. agalactiae*, AdcR also regulates the expression of genes comprising the Adc/Lmb permease (28), but its broader regulon has not yet been determined.

In this study, the global response of *S. agalactiae* to both Zn starvation and excess was investigated by transcriptomics. These results show that the primary adaptive response of *S. agalactiae* to Zn limitation was predominantly mediated by AdcR. The AdcR regulon was then mapped using a combination of transcriptional-fusion assays and *in silico* analyses. This revealed previously unreported AdcR-regulated genes involved in bacterial growth in Zn-restricted conditions including the ribosomal protein, RpsNb, and an ABC transporter.

## RESULTS

### Identification of Zn-regulated genes in *S. agalactiae*

To elucidate how *S. agalactiae* regulates Zn homeostasis, a comprehensive analysis of its global transcriptional response to Zn restriction and intoxication was conducted. The wild-type (WT) *S. agalactiae* A909 strain was grown in chemically defined medium (CDM) in three conditions: (i) without Zn supplementation (Zn-restricted CDM); (ii) 10 µM ZnSO_4_ supplemented medium (Zn-replete CDM); and (iii) 300 µM ZnSO_4_ supplemented medium (Zn-excess CDM). Bacterial growth in the three media was monitored spectrophotometrically by optical density at 600 nm (OD_600_), with only the Zn-excess CDM showing a minor growth delay, relative to the Zn-restricted and Zn-replete conditions (Fig. S1). RNA was isolated from bacteria during logarithmic phase growth, defined as OD_600_ of 0.4, and analyzed by RNA sequencing.

**Fig 1:**
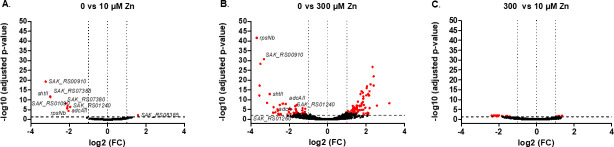
Transcriptomic analysis of genes regulated by Zn concentration in *S. agalactiae* volcano plot of the *S. agalactiae* A909 strain’s transcriptome at mid-exponential phase comparing bacteria grown in (**A**) Zn-restricted CDM (0 µM Zn) vs Zn-replete CDM (10 µM ZnSO_4_), (**B**) Zn-restricted CDM (0 µM Zn) vs Zn-excess CDM (300 µM ZnSO_4_), and (**C**) Zn-excess CDM (300 µM Zn) vs Zn-replete CDM (10 µM ZnSO_4_). Each dot represents one of the A909 genes with its RNA-seq fold change (FC) and adjusted *P*-value calculated from three independent replicates. Red dots are genes differentially expressed (|log2 FC| > 1 and < −1; adjusted *P*-value < 0.05) with selected gene names highlighted. Black dots symbolized non-significant differentially transcribed genes.

Differential gene transcription analyses revealed 10 genes with significant expression changes (fold change ≥ 2; adjusted *P*-value ≤ 0.05) between Zn-restricted CDM and Zn-replete conditions (Fig. 1A; Table S1). This number increased to 220 genes with differential expression between the Zn-restricted and Zn-excess conditions. (Fig. 1B; Table S2). Comparison between Zn-excess and Zn-replete conditions identified differential expression of 29 genes (Fig. 1C; Table S3). The differentially expressed genes were categorized based on their predicted function according to NCBI or Uniprot databases.

Genes differentially expressed in *S. agalactiae* grown in Zn-restricted conditions, as compared to Zn-replete conditions, were commonly associated with the primary adaptive response to Zn deficiency. This includes upregulation of the genes encoding components of the Adc/Lmb transporter (*adcAII*, *shtII*) (Fig. 1; Table S1) (18) and one of the genes associated with a nickel/peptide-permease (SAK_RS01240), which may suggest a role as a secondary Zn importer. The transcript levels of *rpsNb* (*sak_RS08945*) and *sak_RS00910* were also significantly increased during Zn limitation (Table S1). The *sak_RS00910* gene is annotated as encoding a putative metal homeostasis protein, but its function is unknown. The *rpsNb* gene encodes a Zn− form subunit of 30S ribosomal protein S14. This is consistent with the expectation that one or more ribosomal proteins would switch from their Zn+ to the Zn− paralogue (19, 21, 22). By contrast, only one gene (*sak_RS08385*) was significantly downregulated under Zn-restricted conditions, a putative aquaporin of the family of glycerol uptake facilitator proteins (Table S1).

In the comparison between the Zn-replete and Zn-excess conditions, which corresponds to the primary adaptive response to Zn excess, only seven genes were induced in response to Zn stress, mainly ribosomal genes (Table S3). These genes present a fold change just above the threshold which may results from inherent variability in the ribosomal RNA depletion conducted prior to the sequencing. However, a putative aquaporin (SAK_RS08750) of the same family as the aforementioned aquaporin SAK_RS08385 was overexpressed in response to Zn-stress. This may suggest a link between Zn homeostasis and central carbon metabolism, which has previously been reported in *S. pyogenes* (30).

### The primary adaptive response of *S. agalactiae* to Zn starvation is predominantly mediated by AdcR

In the streptococci, the metalloregulator AdcR is the primary mediator of the adaptive response to Zn limitation (26, 28, 29, 31, 32). The *S. agalactiae* A909 genome was investigated to identify putative AdcR binding sites (5′-TTAACNNGTTAA-3′; one mismatch allowed) within intergenic regions of open reading frames. Bioinformatics analyses identified 31 putative sites, 18 of which were within the promoter regions of genes observed in the RNAseq analysis, including nine genes apparently repressed by Zn observed in the comparison of Zn-restricted to Zn-replete media conditions (Table S6; Table S1). AdcR-binding site locations, sequences, and proximity to promoter elements were then analyzed (Table S6). This analysis revealed that most genes had one or more AdcR site(s) situated upstream, a few nucleotides (up to 8) downstream, and/or overlapping the core promoter. Transcriptomic data indicated that these genes were predominantly repressed by increases in cellular Zn. This can be attributed to AdcR binding to the operator sequences and hindering RNAP-promoter interactions, thereby impeding gene transcription. This analysis also identified a putative AdcR-binding site in the promoter of *adhP*, a gene previously reported to be induced in the presence of Zn by AdcR in *S. pneumoniae* and *S. pyogenes* (28, 33) (Table S2; Table S6).

**Fig 2:**
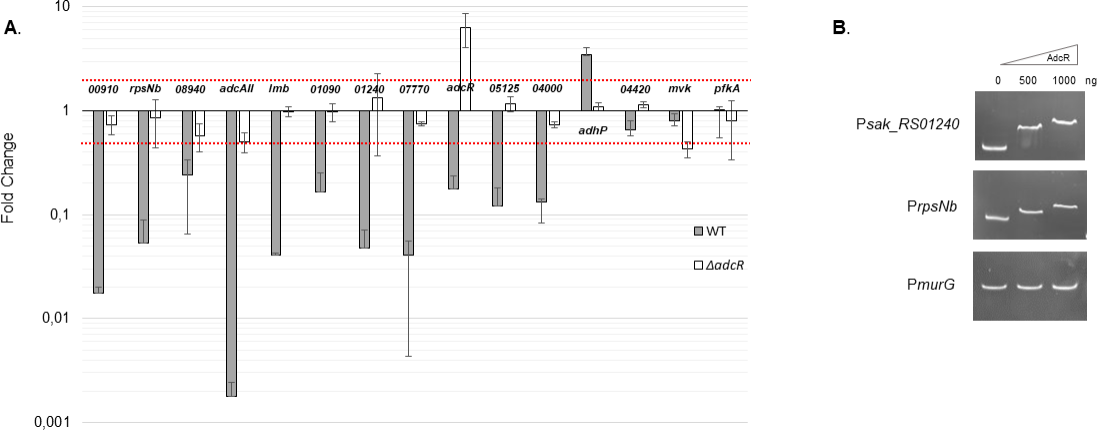
AdcR-dependent regulation of selected genes. (**A**) *S. agalactiae* A909 (black bars) or its isogenic Δ*adcR* mutant (white bars) was grown to mid-log phase (OD_600_ = 0.4) in Zn-restricted CDM or inZn-excess CDM. Expression of *sak_RS00910*, *rpsNb*, *sak_RS08940*, *adcAII*, *lmb*, *sak_RS01090*, *sak_RS01240*, *sak_RS07770*, *adcR*, *sak_RS05125, sak_RS04000, adhP, sak_RS04420, mvk,* and *pfkA* was assessed either by quantitative real time-PCR (qRT-PCR) or by *lacz* transcriptional fusions. Gene expression in Zn-excess CDM in the WT and in the Δ*adcR* mutant strains are presented as a fold change compared to expression of the WT strain grown in Zn-restricted CDM. Red dots delimit significant fold changes (|FC| > 2 and < −0,5). The results are means ± the standard deviations from three independent experiments. (**B**) AdcR-DNA interactions as assessed by gel mobility shift assay. DNA fragments of similar size were amplified from the promoters of *sak_RS01240* (P*sak_RS01240*), *rpsNb* (P*rpsNb*), and the non-specific control promoter of *murG* (P*murG*). Increasing concentrations of AdcR (500 and 1,000 ng) were incubated with the indicated DNA fragments and reaction mixtures were analyzed on a 10% native PAGE.

To further investigate observations from the RNA sequencing analyses and AdcR-dependent regulation, the expression of selected genes in the WT and an Δ*adcR* mutant strain were studied using either quantitative reverse-transcriptase PCR (qRT-PCR) or transcriptional *lacZ* fusion approaches. Here, gene expression profiles were assessed in Zn-restricted and Zn-excess conditions. The data show that the transcriptomic results were largely validated, with *sak_RS00910*, *rpsNb*, *sak_RS08940*, *adcAII*, *sak_RS01090, sak_RS01240*, *sak_RS07770, adcR* and *adhP* demonstrating Zn-dependent expression mediated by AdcR (Fig. 2A). This approach also indicated that the putative AdcR box within the promoter of *sak_RS04420* and of *mvk* (*sak_RS06830*) genes did not exert Zn- or AdcR-dependent regulation of gene expression.

The targeted transcriptional analyses did identify some discrepancies with respect to the global transcriptomic analyses. Notably, in the RNA sequencing analyses, no upregulation of the *lmb-sht* operon was observed, despite established Zn-dependent gene expression and the presence of two AdcR binding sites (18). Consistent with that prior report, qRT-PCR experiments show that *lmb* expression was regulated by Zn and AdcR (Fig. 2A). Expression of the *sak_RS05125* and *sak_RS04000* genes, which encode a putative pneumococcal-type histidine triad protein and a hypothetical protein, respectively, was also determined to be regulated by Zn and AdcR consistent with the presence of an AdcR-binding site downstream of their promoter (Fig. 2A; Table S6). Finally, among the 31 genes presenting with at least one AdcR-binding site within their promoter regions, further experiments validate that the *S. agalactiae* AdcR regulon comprises at least 17 genes or operons (Table S6).

To further interrogate AdcR-dependent gene regulation, two AdcR-regulated genes, *rpsNb* and *sak_RS01240*, and one AdcR-independent control gene, *murG*, were investigated. Here, the direct and specific binding of AdcR to the promoter region of these genes was assessed using electrophoretic mobility shift assays (Fig. 2B). The data show that increasing concentrations of recombinant AdcR were associated with a shift in the electrophoretic mobility of DNA fragments containing the promoter regions of *sak_RS01240* and *rpsNb*. By contrast, DNA fragments containing the promoter region of *murG* were unaffected, consistent with its lack of an AdcR-binding site.

### Three AdcR binding sites are involved in the Zn-dependent repression of *rpsNb*

**Fig 3:**
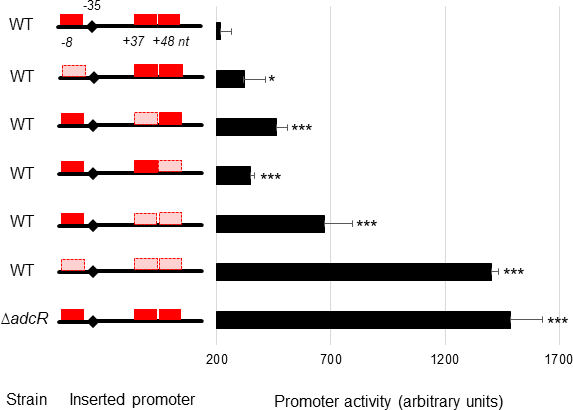
The three putative AdcR binding sites within the *rpsNb* promoter are involved in full Zn-dependent promoter repression. Transcriptional *lacZ* fusions with either the native *rpsNb* promoter region or the *rpsNb* promoter region containing point mutations destroying each putative AdcR-box (isolated and combined mutations) were constructed and introduced into the WT strain. The native *rpsNb* promoter region was also introduced in a Δ*adcR* mutant strain. The dark red boxes represent native AdcR-boxes, whereas light red represents mutated AdcR-boxes. β-Galactosidase assays were performed as described in Materials and Methods. The activity of the promoters was measured in Zn-excess CDM (300 µM of ZnSO_4_) (black bars). The values shown are the means ± standard deviations of three independent assays. The asterisks indicate *P* values obtained using an unpaired Student *t* test compared to the promoter activity of the native *rpsNb* promoter region in the WT strain. *P < 0.05; **P < 0.01; ***P < 0.001. The dark red rectangle indicates native AdcR box and the light red rectangle indicates a mutated AdcR box. The position of the predicted −35 hexamer is indicated by a diamond.

Building on the observation of AdcR-mediated regulation of *rpsNb*, further analysis of the promoter region of the gene revealed three putative AdcR binding sites, henceforth named as Box 1 (8 nt upstream of the −35 promoter element), Box 2, and Box 3 (overlapping AdcR boxes located downstream of the −10 promoter element). Notably, each of the three putative AdcR boxes contained a mismatch compared to the consensus sequence (5′-TTAACNNGTTAA-3′) (Table S6; Fig. 3). The role of these AdcR boxes in *rpsNb* expression was then investigated by combining mutagenesis of the promoter region of *rpsNb*, wherein a point mutation was introduced to disrupt the palindromic operator sequence, with transcriptional *lacZ* fusions (Fig. 3). The data show that mutation of any AdcR-binding site impaired Zn-dependent repression of *rpsNb* promoter, relative to the wild-type sequence. All three AdcR boxes exert Zn-dependent transcriptional control over *rpsNb* expression, albeit to differing extents. These differences may be attributable to deviations from the consensus sequence for AdcR binding sites. Furthermore, the data show that AdcR regulation of *rpsNb* expression was mediated in the presence of Zn, but not by other metals (Fig. S2).

**Fig 4:**
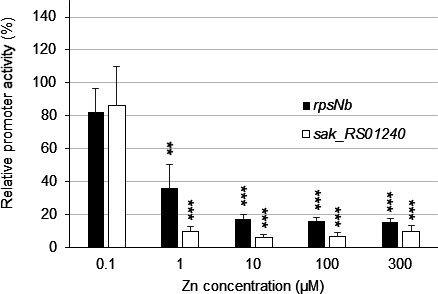
Expression of the *rpsNb* and *sak_RS01240* genes is gradually repressed by Zn. The *rpsNb* and *sak_RS01240* promoter activity was measured in Zn-restricted CDM supplemented with various amounts of added metals (0 to 300 µM). Cells containing the P*_rpsNb_-lacZ* transcriptional fusions were grown until the mid-exponential phase of growth (OD_600_ = 0.4), and β-galactosidase assays were performed as described in Materials and Methods. The reference value (100%) corresponds to the *rpsNb* or *sak_RS01240* promoter activity in Zn-restricted CDM (0 µM Zn). The values shown are mean results ± standard deviations. The asterisks indicate *P* values obtained using an unpaired Student *t* test, comparing the promoter activity of cells grown in Zn-restricted CDM and cells grown in CDM with the various added Zn concentrations. **P < 0.01; ***P < 0.001.

**Fig 5:**
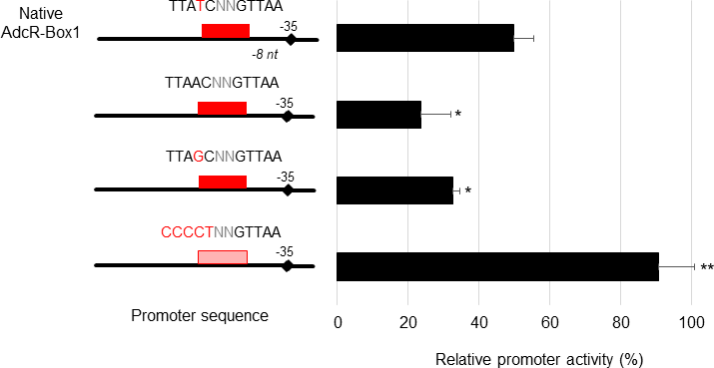
Effect on zinc repression of *rpsNb* of different mismatches in the AdcR box. Transcriptional *lacZ* fusions with either the *rpsNb* promoter region containing only the native AdcR-Box1 or the *rpsNb* promoter region with substitution of selected nucleotide were constructed and introduced into the WT strain. The dark red boxes represent functional AdcR-boxes, whereas the light red box represents inactivated AdcR-box. Bacteria were grown either in Zn-restricted CDM or in CDM supplemented with 300 µM Zn until the mid-exponential phase of growth (OD_600_ = 0.4). β-Galactosidase assays were performed as described in Materials and Methods. Results are expressed as a relative promoter activity with the reference value (100%) corresponding to the promoter activity in Zn-restricted CDM. The values shown are the means ± standard deviations of three independent assays. The asterisks indicate *P* values obtained using an unpaired Student *t* test between the promoter activity of the native AdcR-Box1 compared to AdcR boxes carrying mismatches. *P < 0.05; **P < 0.01.

Relative promoter activities of *rpsNb* and *sak_RS01240* were then analyzed. In contrast to *rpsNb*, the promoter region of *sak_RS01240* contains a single consensus AdcR-binding sequence located 4 nucleotides upstream of its −35 promoter element. Introduction of a point mutation in the *sak_RS01240* AdcR-binding site abrogates Zn-dependent gene expression (Fig. S3). Similar to *rpsNb*, the gene *sak_RS01240* showed substantial repression in 1 µM ZnSO_4_ supplemented media and maximum repression in 10 µM ZnSO_4_ supplemented media (Fig. 4). In our conditions, the presence of three AdcR boxes within the *rpsNb* promoter region did not increase either the sensitivity to Zn or the fold-repression compared to *sak_RS01240*, which has only one AdcR box. Nevertheless, the impact of the observed mismatches in the AdcR binding sites of *rpsNb* warranted further investigation. To that end, the first *rpsNb*-binding site (Box 1) was “repaired” and we observed that repression of *rpsNb* by Zn is 50% more efficient when the AdcR-binding site is a perfect palindromic sequence (Fig. 5). The two overlapping AdcR sites, Boxes 2 and 3, also share a A →T mismatch in the first part of each consensus sequence and we speculate that it also contributes to reduced transcriptional regulation of AdcR, most likely due to reduced binding affinity. This could explain why, despite three AdcR binding sites within its promoter, the *rpsNb* gene is not significantly more repressed in the presence of Zn than genes containing only one. Using this framework, the introduction of an A→G substitution at nucleotide 4 (5′-TTAGCNNGTTAA-3′), as occurs in the gene *sak_RS04000* (Table S6), resulted in a reduced efficiency of AdcR-mediated repression (Fig. 5). This observation is consistent with *sak_RS04000* showing regulation by Zn and AdcR (Fig. 2).

### Expression and conservation of the duplicated ribosomal proteins RpsNa and RpsNb

**Fig 6:**
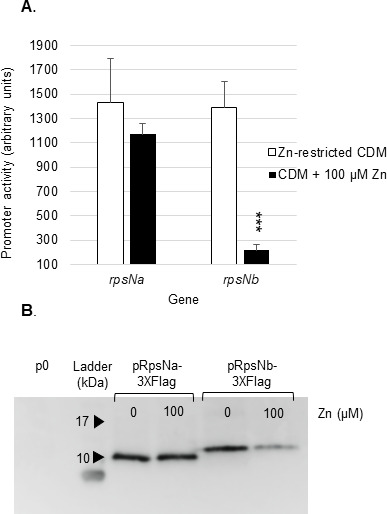
Comparative expression of the RpsNa and RpsNb proteins. Cells containing the *lacZ* transcriptional fusions or proteins fused to the 3×Flag were grown until the mid-exponential phase of growth (OD_600_ = 0.4) in Zn-restricted CDM or in CDM supplemented with 100 µM of Zn. (**A**) The *rpsNa* and *rpsNb* promoter activity was measured by β-galactosidase assays. The values shown are mean results ± standard deviations. The asterisks indicate *P* values obtained using unpaired Student’s *t* test, comparing promoter activity of cells grown in Zn-restricted CDM and cells grown in CDM with 100 µM added Zn concentrations. ***P < 0.001. (**B**) Expression of RpsNa-3×FLAG and RpsNb-3×FLAG of an expected size of 9.77 and 13.2 kDa, respectively. Protein extract from *S. agalactiae* with empty plasmid (left lane) was used as a specificity control. Immunoblot analysis was performed with anti-Flag antibody. Brilliant Blue staining was done as a protein loading control.

Prokaryotes frequently encode Zn-dependent and Zn-independent ribosomal proteins, henceforth referred to as Zn+ and Zn− forms, respectively (19, 21). In *B. subtilis*, during growth in Zn-limiting conditions, the metalloregulator Zur, an ortholog of *S. agalactiae* AdcR, facilitates expression of the Zn− form ribosomal proteins. We investigated the *rpsNb* gene, potentially encoding 30S ribosomal protein S14, as a putative ribosomal paralogue regulated by Zn *via* AdcR. Bioinformatic analysis on *S. agalactiae* A909 genome, identified RpsNa (SAK_RS00515) as a potential Zn+ form paralogue of RpsNb. Alignment of RpsNb and RpsNa showed that both proteins are very similar (51% sequence identity over 61 amino acids) and that RpsNa contains two Zn-ribbon motifs (CxxC) that are absent in RpsNb (Figure S4A). Structural comparisons of RpsNa with RpsNb indicated that the two proteins had similar overall folds (Figure S4B and C). We hypothesize that RpsNa is the Zn+ form of the S14 protein, and RpsNb is its Zn− form paralogue regulated by a Zn-dependent repressor. Expression of the RpsNa/RpsNb pair was examined and compared both at the transcriptional and at the protein level, using respectively plasmids containing *rpsNa* and *rpsNb* promoter region fused to *lacZ* or plasmids expressing RpsNa and RpsNb fused to a 3×FLAG tag. Bacteria were grown in either Zn-restricted CDM or CDM supplemented with 100 µM ZnSO_4_, a concentration ensuring maximum Zn-repression without impairing growth. As shown in Fig. 6, RpsNa expression is high and constant in the tested conditions, whereas RpsNb is repressed by Zn. Notably, no significant RpsNa degradation was observed in Zn-restricted CDM, indicating that the rapid protein degradation model, to liberate protein-bound Zn during Zn deficiency, did not occur under our experimental conditions.

Analysis showed that *rpsNa* and *rpsNb* loci are highly conserved (100% identity at the nucleotide level) in the 218 *S*. *agalactiae* strains whose complete genome sequences were available in the NCBI database. Among the main pathogenic *Streptococcus* or closely related pathogenic bacterial species, S14 ribosomal proteins are mostly duplicated (or even triplicated in the case of *Enterococcus faecalis*) and a candidate binding site for Zn repressor (Zur or AdcR) is found in the promoter region of the Zn− form paralog (Table 1). The one exception is *S. pneumoniae*, which encodes only a Zn− form of S14 with no candidate binding site for a Zn repressor in its promoter.

**TABLE 1:** Duplication of S14 ribosomal proteins in pathogenic cocci^*a*^

Bacteria	S14 (RpsN)^*b*^	
	Zn+ form (RpsNa)	Zn− form (RpsNb)
*Streptococcus agalactiae*	+	+ (3 AdcR box)
*Streptococcus pyogenes*	+	+ (2 AdcR box)
*Streptococcus pneumoniae*	−	+
*Enterococcus faecalis*	+	+ (1 Zur box) + (1 Zur box)
*Staphylococcus aureus*	+	+ (1 Zur box)

^*a*^ Protein homologs were searched by Blast-P analysis using RpsNa and RpsNb proteins of *S. agalactiae* A909 as queries against species genomes available in the NCBI database.

^*b*^ Candidate zinc repressor binding sites, defined by an AdcR or Zur box, in the upstream region of the genes with presence denoted by “+” and absence denoted by “−.”

### RpsNb is important for *S. agalactiae* growth in Zn-restricted environments

**Fig 7:**
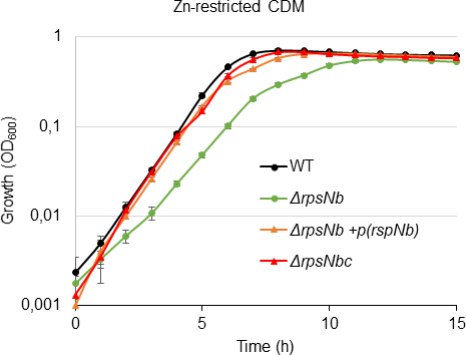
RpsNb is important for bacteria growth in Zn-restricted CDM. The *S. agalactiae* WT, Δ*rpsNb* strains and the two complemented strains Δ*rpsNb* + *P*(*rpsNb*) (plasmidic pTcvP*tet* complementation) and Δ*rpsNb^c^* (chromosomic complementation) were grown in Zn-restricted CDM (0 µM ZnSO_4_). The empty vector pTcvP*tet* (ᴓ) was inserted in the WT, Δ*rpsNb,* and Δ*rpsNb^c^* strains as a control. Growth was monitored by OD_600_ measurements every 30 min for 15 h. The data are presented as mean OD_600_ measurements ± the standard deviations from three independent experiments.

Prior studies of Zn limitation in *B. subtilis* have suggested that expression of the Zn− form paralogues of the ribosomal proteins enables the restricted cellular Zn pool to be redistributed, thereby promoting bacterial adaptation and survival (22, 34). Here, the contribution of RpsNb in bacterial survival in Zn-limited conditions was addressed by generating a mutant strain lacking RpsNb. We were unable to obtain a Δ*rpsNa* strain, which aligns with findings from a study using a Krmit transposon (Tn) mutant library in the CJB111 *S. agalactiae* strain, indicating that *rpsNa* is an essential gene in THY-rich medium (16). Growth experiments in Zn-restricted CDM show that RpsNb has a crucial role in supporting optimal growth in these conditions as the *S. agalactiae* Δ*rpsNb* strain displayed a substantially growth delay relative to the WT strain (Fig. 7). This growth defect was specific to Zn-limited conditions as there was no apparent difference in growth rates when cultured in rich TH medium or in Zn-replete CDM (10 µM ZnSO_4_) (Fig. S5).

The *S. agalactiae* Δ*rpsNb* strain was complemented either using the pTCV-Ptet *rpsNb* plasmid, allowing ectopic expression of *rpsNb* under the control of a constitutive promoter, or by *in situ* chromosomal complementation. Both complemented strains presented a restored growth (Fig. 7).

**Fig 8:**
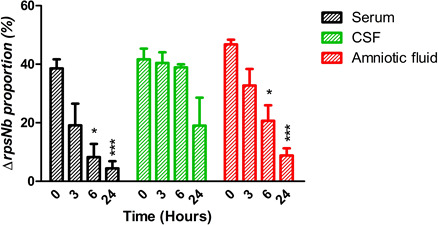
The RpsNb ribosomal protein participates in *S. agalactiae* survival in human biological fluids. A909 WT and Δ*rpsNb* mutant strains were inoculated in equivalent numbers into human serum, cerebrospinal fluid (CSF), or amniotic fluid. Cocultures were incubated at 37°C for 24 h without agitation. The proportion of each strain was monitored by plating diluted cultures on TH agar containing erythromycin (10 µg mL^−1^) and X-Gal (60 µg mL^−1^) (see Materials and Methods). Results are presented as the means ± standard deviations for three independent cocultures. The asterisks indicate *P* values obtained using an unpaired Student *t* test to compare the proportion of the strain at T0 and its proportion at the indicated times. *P < 0.05; **P < 0.01; ***P < 0.001.

During infection, *S. agalactiae* transitions through numerous anatomical niches wherein it encounters varying levels of Zn bioavailability. For example, in serum, Zn concentration can exceed 14 µM but it is complexed with molecules such as albumin and alpha-2-macroglobulin (9, 35). To further investigate the role of RpsNb in bacterial propagation and adaptation to human biological fluids, a competition assay between the *S. agalactiae* WT and Δ*rpsNb* strains was conducted. This showed that, over a period of 24 h, the WT strain was able to outcompete the Δ*rpsNb* mutant in all biological fluids, with the decreases in the plasma and amniotic fluid being statistically significant at 6 and 24 h (Fig. 8).

Taken together, these results indicate that RpsNb contributes to bacterial survival in human biological fluids *in vitro* and further highlight that bacterial adaptation to Zn bioavailability is a key component of the host-pathogen interaction.

### Identification of a potential secondary Zn transporter in *S. agalactiae*

Among the nine genes repressed by Zn, when comparing Zn-restricted compared to Zn-replete conditions, constituting the first adaptive responses of *S. agalactiae* to Zn limitation, we identified the *sak_RS01240* gene. This gene encodes an ABC transporter solute-binding protein belonging to the peptide/opine/nickel uptake (PepT) family. Transporters of this family have been shown to enable the uptake of nickel, cobalt, zinc, copper, or iron in *S. aureus* as metal-chelate complexes (15, 36–39). An AdcR-binding site was identified within the promoter region of the *sak_RS01240-RS01260* operon, which encodes a complete ABC permease comprising a putative cluster C solute binding protein (SBP; *sak_RS01240*), the transmembrane domains (*sak_RS01245* and *sak_RS01250*), and the cytoplasmic nucleotide-binding domains (*sak_RS01255* and *sak_RS01260*). Transcriptomic analyses show that expression of the SBP and nucleotide-binding domains are transcriptionally regulated by Zn (Table S2), with transcriptional fusion assays demonstrating that this regulation occurs via AdcR for *sak_RS01240* (Fig. 2).

**Fig 9:**
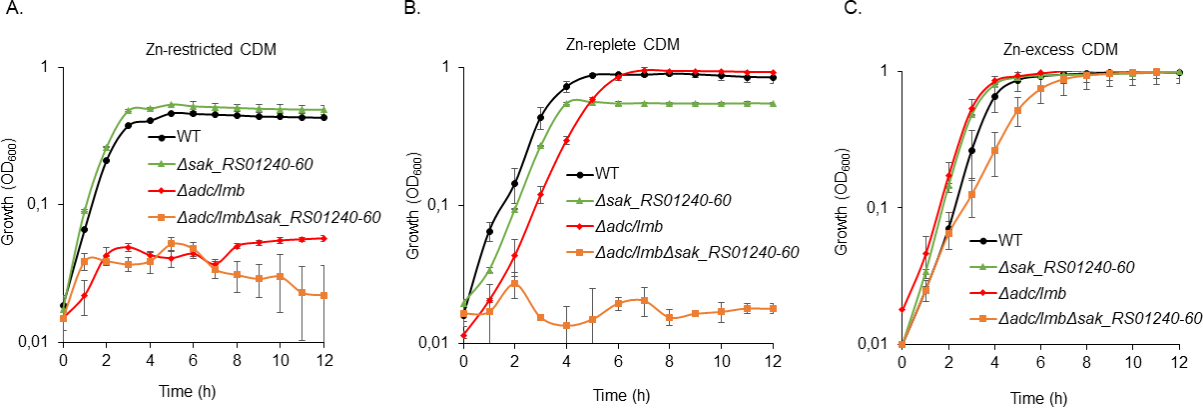
SAK_RS01240-RS01260 is a potential secondary Zn-transporter in *S. agalactiae.* The *S. agalactiae* WT, ∆*sak_RS01240-RS01260*, ∆*adc/lmb* single mutant, and ∆*adc/lmb* ∆*sak_RS01240-RS01260* double mutant strains were grown overnight in CDM with 25 µM ZnSO_4_ and inoculated at an OD_600_ of 0.005 either in Zn-restricted (0 µM ZnSO_4_) (**A**), Zn-replete CDM (10 µM ZnSO_4_) (**B**), or Zn-excess (300 µM ZnSO_4_) (**C**) conditions. Growth was monitored by OD_600_ measurements every hour for 12 h. The data are presented as mean OD_600_ measurements ± the standard deviations from three independent experiments.

To evaluate the relative contribution of this system to cellular Zn accumulation, we constructed single and combination mutant variant strains of the primary Zn uptake pathway, the Adc/Lmb ABC permease (∆*adc/lmb*) (18), and the SAK_RS01240-RS01260 system (∆*sak_RS01240-RS01260*). In all tested conditions, the ∆*sak_RS01240-RS01260* strain behaved similarly to the WT strain, whereas the ∆*adc/lmb* mutant was unable to grow in Zn-restricted conditions (Fig. 9). These data support the essentiality of the Adc/Lmb ABC permease in *S. agalactiae* Zn acquisition. In Zn-replete conditions (10 µM ZnSO_4_), the ∆*adc/lmb* strain showed growth comparable to the parental and ∆*sak_RS01240-RS01260* strain, while the combination strain (∆*adc/lmb*∆*sak_RS01240-RS01260*) was unable to grow. This growth defect was largely abrogated upon further supplementation of Zn (100 µM ZnSO_4_) indicating that impaired Zn uptake was the most probable basis for the phenotypic impact. Moreover, growth of ∆*adc/lmb*∆*sak_RS01240-RS01260* strain in Zn-replete conditions was also restored in the *sak_RS01240-RS01260* complemented strain (Fig. S6). Taken together, these data support a role for the SAK_RS01240-RS01260 in cellular Zn accumulation, most likely as a potential secondary Zn transport system. However, further experiments are required to define how the SBP, SAK_RS01240, recruits metal ions, that is as cations or metal-chelate complexes, and its specificity.

## DISCUSSION

This study reports the global transcriptome changes in *S. agalactiae* in response to Zn availability. Differential gene transcription analysis uncovered 10 genes with significant expression changes between Zn-restricted CDM and Zn-replete conditions, 29 genes between Zn-replete and Zn-excess conditions, and 220 genes between Zn-restricted and Zn-excess conditions. Among the genes differentially expressed when comparing Zn-restricted to Zn-replete conditions, 90% exhibit an AdcR box (5′-TTAACNNGTTAA-3′; one mismatch allowed) in their promoter region and are repressed by Zn. Complementary experiments revealed that the primary response to Zn limitation in *S. agalactiae* is predominantly mediated by AdcR. By contrast, the 29 genes that were differentially expressed upon Zn intoxication, revealed in the transcriptomic comparisons of Zn-replete and Zn-excess media, lacked AdcR-binding sites. This indicates that they are regulated independently of AdcR, most likely by SczA which has been studied in other works (40). However, some of our findings differ from those of this study, which identified 567 genes differentially expressed in response to Zn stress including the *czcD* efflux system, which is the major pathway for Streptococcal resistance to Zn intoxication. We did not observe upregulation of *czcD* in response to Zn excess in either our RNA seq data or in qRT-PCR analysis under our experimental conditions (data not shown). A key distinction between the studies is that we employed CDM restricted in Zn, as well as in other metals, supplemented with 300 µM of Zn, whereas the other study used a rich TH medium supplemented with 250 µM of Zn. This may suggest that *czcD* expression is influenced not only by Zn concentration but also by the presence of other metals. Alternatively, it may be that *czcD* expression was not significantly induced under our experimental conditions due to intracellular Zn being buffered on small molecules, such as glutathione, and/or Zn-dependent metalloproteins, such as ribosomal proteins.

*In silico* analyses of the AdcR box palindromic sequence (one mismatch allowed) within the *S. agalactiae* A909 genome identified 31 open reading frames harboring a potential AdcR box in their promoter region. Of these, 19 putative genes were observed in our transcriptomic analyses as potentially Zn-regulated (Table S6). Further analyses of the AdcR binding sites revealed that alteration of the palindromic sequence, via one A ↔ T or A ↔ G exchange, impairs AdcR-mediated regulation but does not abolish it. Only 17 of the putative operons had functional AdcR box(es) within their promoter, further refining the designation of the *S. agalactiae* AdcR regulon.

The AdcR regulon includes the Zn-specific Adc/Lmb permease, the 30S ribosomal protein S14 RpsNb, and the alcohol dehydrogenase AdhP. This inference is consistent with prior studies of *S. agalactiae* and other streptococci (17, 18, 28, 29, 32, 33, 41). Furthermore, we identified new targets of the AdcR regulon including a gene encoding a protein of unknown function (*sak_RS00910*); genes encoding two ABC permeases of the peptide/opine/nickel uptake (PepT) family; a potential ammonium transporter (*sak_RS07375*); a putative flavoprotein involved in potassium transport (*sak_RS07380*); a putative rhodanese-related sulfur-transferase (*sak_RS07385*), a putative DNA/RNA endonuclease (*sak_RS01090*); a putative LPXTG cell wall anchor domain-containing protein (*sak_RS04480*); a putative histidine triad protein (*sak_RS05125*); and putative proteins of unknown function (*sak_RS08940* and *sak_RS04000*). While all these genes are, by their AdcR-mediated regulation, implicated in *S. agalactiae* adaptation to Zn limitation, the underlying roles and contributions for many remain to be defined. It is also important to highlight that their contribution may be related to resistance within an *in vivo* context of poor Zn bioavailability. Thus, their roles may contribute to survival during exposure to nutritional immunity and aid in subverting the innate immune response. In addition, they may also aid in survival within Zn-deficient hosts. Dietary Zn deficiency affects approximately 80% of pregnant women worldwide and is associated with increased levels of *S. agalactiae* vaginal colonization (42).

Among the genes containing an AdcR box within their promoter, only *adhP* is upregulated in the presence of Zn. In this case, the AdcR box was positioned 31 nt downstream of the core promoter (Table S6; Fig. S7). A similar situation is observed in *S. pneumoniae*, where an AdcR Box is positioned 29 nt downstream of the core promoter of *adhP* and direct binding of AdcR within the *adhP* promoter has been demonstrated (28). In these configurations, the hypothesis that AdcR could directly activate *adhP* transcription by recruiting/stabilizing the binding of RNA polymerase seems unlikely. This is because such mechanisms require the binding of the regulator immediately upstream or downstream of the promoter elements, with activator-dependent promoters generally lacking recognizable −10 or −35 motifs, which is not the case for *adhP*. Therefore, these data suggest that the binding of AdcR within the *adhP* promoter may serve to impair the activity of another repressor by steric occlusion. The identity of this alternative regulatory protein remains to be defined. Similar mechanisms of activation have been reported in *S. pyogenes*, where in addition to *adhP*, AdcR was shown to mediate upregulation of the *hasABC* operon encoding the enzymes involved in capsule biosynthesis, and *prtS* that encodes for a cell envelope-bound membrane protease in the presence of Zn (33). For these latter genes, AdcR was shown to bind to a novel non-canonical DNA-binding motif. Here, it has been proposed that AdcR prevents CovR, a major streptococcal virulence-associated regulator, from binding to the *hasA* promoter and thus promotes transcription activation by relieving CovR-mediated repression (33). Although no homologues of *hasABC* and *prtS* were found in *S. agalactiae*, we cannot exclude that in this bacterium. AdcR also possesses a non-canonical DNA-binding motif, which could further extend its regulon.

In this study, we investigated the involvement of the ribosomal protein RpsNb in *S. agalactiae*’s adaptation to Zn deprivation. We hypothesized that RpsNb represents the Zn− form of the 30S ribosomal protein S14, RpsNa. Under our tested conditions, we observed Zn-mediated AdcR regulation of RpsNb, whereas RpsNa expression remained constant and high. Moreover, RpsNb is important for *S. agalactiae* growth in Zn-restricted conditions. In a model where switching from Zn+ to Zn− paralogues of ribosomal proteins leads to the degradation of the Zn+ form to release Zn for other cellular functions, we expected to observe RpsNa degradation under Zn-restricted conditions. However, our immunoblot analysis did not show any RpsNa degradation. Confirmation of changes in the relative abundance between RpsNa and RpsNb within *S. agalactiae* ribosomes in Zn-depleted or in Zn-replete conditions could provide further insights into how RpsNb participates in resistance to Zn deprivation.

The SAK_RS01240-RS01260 system, which belongs to the peptide/opine/nickel uptake (PepT) family, was identified as a potential secondary Zn transport system in this work. In *S. aureus*, two transporters of the same family are involved in the uptake of nickel or nickel/cobalt/copper/iron, respectively (15, 37–39). In *S. agalactiae*, we did not observe any improved growth of the ∆*sak_RS01240-RS01260* or ∆*adc/lmb* ∆*sak_RS01240-RS01260* mutant strains after the addition of nickel, cobalt, copper, or iron in the medium (data not shown), and these metals had no effect on the *sak_RS01240* expression, contrary to Zn. However, further investigations are needed to identify more precisely the substrate specificity of the SAK_RS01240-RS01260 system and whether it acquires metal ions directly or via a chelate complex.

Despite a significant growth delay in Zn-restricted conditions, the ∆*adc*/*lmb* ∆*sak_RS01240-RS01260* mutant grew similarly to the WT strain in the presence of 100 µM Zn and rich media. This indicates that in conditions of high Zn bioavailability, *S. agalactiae* A909 acquires sufficient Zn via other uptake pathways, that is, low affinity or non-specific systems. One possible alternative pathway is the SAK_RS07770-RS07790 ABC transporter. This system has an AdcR box located within its promoter region and, similar to the SAK_RS01240-RS01260 system, belongs to the PepT family of transporters. This inferred role in Zn homeostasis is supported, at least in part, by a recent mariner transposon (Krmit) mutant library study of *S. agalactiae* that sought to identify genes involved in resisting metal restriction mediated by calprotectin exposure. Here, loss of the *adc*/*lmb* and SAK_RS01240-RS01260 system or the SAK_RS07770-RS07790 system, resulted in a fitness defect in the presence of subinhibitory calprotectin concentrations (16). Collectively, these findings indicate that *S. agalactiae* A909 uses multiple transporters to achieve Zn sufficiency, with the molecular basis of metal acquisition via the PepT-type transporters warranting further investigation.

## MATERIALS AND METHODS

### Bacterial strains and culture conditions

*E. coli* XL1-Blue served as the host for recombinant plasmid pG+host1^TS^, pTCV-PTet, and pTCV-*lac. E. coli* strains were cultured on Luria-Bertani (LB) agar plates or in liquid LB medium at 37°C with agitation (200 rpm). *E. coli* strains harboring pG+host1^TS^, pTCV-*lac,* or pTCV-PTet plasmids were maintained in medium containing 150 µg mL^−1^ of erythromycin (Ery). *E. coli* strains harboring pET28a plasmid were maintained in medium containing 25 µg ml^−1^ of kanamycin and chloramphenicol.

*S. agalactiae* A909 were cultured on 5% (vol/vol) horse blood Trypticase soy agar plates (1.5% agar; BioMérieux) on Todd-Hewitt broth (TH) agar (Sigma-Aldrich) or in liquid TH medium at 37°C without agitation. Strains harboring pG+host1^TS^, pTCV-*lac,* or pTCV-PTet plasmids were maintained in medium containing 10 µg mL^−1^ of Ery. *S. agalactiae* strains used throughout this study are listed in Table S4.

### *S. agalactiae* culture in chemically defined medium

*S. agalactiae* strains were cultured in Zn-restricted chemically defined medium (CDM) as previously described (18). Briefly, *S. agalactiae* was grown in TH until it reached stationary phase and was then inoculated, at an optical density at 600 nm (OD_600_) of 0.005, into Zn-restricted CDM supplemented with 0.2% (wt/vol) glucose [d-(+)-glucose; Sigma-Aldrich] and 500 µM ethylenediaminetetraacetic acid (EDTA) to chelate residual metals, and grown overnight. *S. agalactiae* was then inoculated at an OD_600_ of 0.005 into Zn-restricted CDM supplemented with 1% glucose (wt/vol), 500 µM EDTA, and ZnSO_4_, MnCl_2_, FeSO_4_, or CuSO_4_ at indicated concentrations. *S. agalactiae* strains were grown at 37°C for 24 h in microtiter plates containing 200 µL of medium per well (Greiner Bio-One; Cellstar), with OD_600_ measurements recorded using an Eon spectrophotometer (BioTek).

### Construction of deletion mutant and complementation strains

Non-polar deletion of the entire coding sequence of the *rpsNb* and of the *sak_RS01240-RS01260* operon had been generated as previously described using the thermo-sensitive shuttle plasmid pG+host1 and a two-step homolog recombination (18). PCR was carried out with the Platinum SuperFi polymerase (Thermofisher) and primers used to generate mutant strains are listed in Table S5.

Plasmid complementation of the ∆*rpsNb* and ∆*sak_RS01240-RS01260* mutant strains was carried out by PCR amplification of the entire coding sequence of *rpsNb* or gene *sak_RS01240-RS01260* (primers indicated in Table S1) and cloning into the pTCV-P_Tet_ vector using appropriate restriction sites as previously described (18). The cloned sequence into the pTCV-P_Tet_ vector was confirmed by PCR and DNA sequencing. Oligonucleotides (Sigma-Aldrich) used for the complementation constructs are listed in Table S5.

The chromosomic *in situ* complementation was carried out by PCR amplification of the upstream region, the coding sequence, and the downstream region of *rpsNb* (primers indicated in Table S5) and cloning into the pG+host. After the allelic exchange, *in situ* complementation was confirmed by PCR and sequencing.

### RNA extraction and sequencing

Total RNA was extracted from cells grown in CDM to the mid-exponential phase (OD_600_ = 0.5). Cells were harvested (10 mL) and the pellets were frozen and stored at −80°C. Pellets were lysed mechanically with glass beads in a FastPrep-24 instrument. Total RNA was extracted via a phenol/Trizol purification method (43) Concentration and purity of RNA were controlled with a NanoDrop Lite instrument (Thermo Scientific) by analysis of the A260/A280 ratio. RNA products were treated with a DNase (Turbo DNA-free DNase; Ambion) according to the manufacturer’s instructions and the absence of DNA contamination was confirmed by PCR using 50 ng of the purified RNA. RNA quality was validated using the Agilent 2100 Bioanalyzer system.

rRNA depletion and RNA-sequencing were completed by Novogene using the Illumina HiSeq-SE50 platform to generate 150 bp paired-end reads. The raw reads were filtered by Trimmomatic (44) to remove low-quality reads. Trimmed reads were mapped to the *S. agalactiae* A909 genome (GCF_000012705.1) using Bowtie2 (45) with default parameters. Mapped read counts were generated using HTSeq-count (46) and differentially expressed genes were defined using DESeq2 (47). Genes with an adjusted *P*-value of ≤0.05 and fold change ≥2 were considered significantly modulated. Volcano plots of differentially expressed genes were prepared using Graphpad Prism 9 software.

### Reverse transcription and quantitative reverse-transcriptase PCR (qRT-PCR)

One microgram of RNA was reverse transcribed using the iScript cDNA synthesis kit (Bio-Rad) according to the manufacturer’s instructions. qRT-PCR was performed in a 10 µL reaction volume containing 40 ng of cDNA, SybrGreen I master mix (Roche) (1×), and 0.5 µM of primers (Table S1). Amplification and detection were assessed with a LightCycler 480 PCR detection system. The specificity of the amplified product and the absence of primer dimer formation were verified by generating a melting curve (65-95°C in continuous). The crossing point (*C_P_*) was defined for each sample. The expression levels of the tested genes were normalized with respect to 16S rRNA whose levels did not vary under our experimental conditions. Each assay was performed in duplicate and repeated with at least three independent RNA samples.

### Construction of *lacZ* transcriptional fusions and strains expressing RpsNa/b-3×Flag proteins

Plasmid pTCV-*lac* which carries a promoterless *lacZ* gene (48) was used to construct transcriptional lacZ reporter fusions. Promoters were amplified by PCR using primers listed in Table S5 and cloned into the EcoRI/BamHI or EcoRI/SmaI sites.

Site-tagged mutagenesis within the *rpsNb* promoter was performed to obtain the three mutated AdcR-binding sites in which five nucleotides were replaced so as to destroy the palindromic operator sequence without disturbing RNA polymerase fixation. Two DNA fragments were generated by PCR using oligonucleotides containing mismatches (see Table S5) and carrying BsaI restriction sites, a type IIS restriction endonuclease, which cleaves after its restriction site, generating DNA fragments whose tetranucleotide cohesive ends. Site-tagged mutagenesis of the AdcR Box within the *adhP* and *sak_RS01240* promoters was achieved by inserting the substitution into the forward primer (Table S5).

To construct pTCV plasmids expressing RpsNa/b-3×FLAG, the promoter and coding sequence of RpsNa or RpsNb and the coding sequence of 3×FLAG were amplified using primers listed in Table S5. Fragments were ligated after BsaI restriction. The resulting fragments were cloned into the pTCV-lac vector using EcoRI and BamHI restriction sites. The vectors were confirmed by PCR and DNA sequencing.

### Beta-galactosidase assay

Bacteria were grown in 10 mL of CDM medium and harvested (10 mL samples) during the mid-exponential phase of growth (OD_600_ = 0.4). Cells were resuspended in 500 µL of Z buffer (49) supplemented with O.5% DTT and lysed mechanically with glass beads using FastPrep-24 instruments. Cell debris was eliminated by centrifugation (5 min, 6,000 rpm). Protein concentrations were determined using the Bio-Rad protein assay (Bio-Rad, Hercules, CA). The supernatants were used for assays as previously described (49) using o-nitrophenyl-β-D-galactopyranoside (ONPG; 4 mg mL^−1^) and β-galactosidase-specific activities were expressed as arbitrary units/mg protein.

### AdcR purification

The AdcR coding sequence was amplified by PCR using primers OAH496 and OAH497 (Table S5). PCR amplifications were cloned into pET28a (+) vector (EMD Biosciences) in *E. coli* BL21 codon + (DE3)-RIL (Novagen) for high-level expression and addition of an C amino-terminal 6×His tag. Recombinant strains were grown in LB broth to an OD_600_ of 0.5 at 37°C, shaking at 200 rpm in an orbital shaking incubator. Protein expression was induced by the addition of 1 mM of isopropyl-β-D-1-thiogalactopyranoside (IPTG), with cells then grown for a further 5 h at 37°C at 200 rpm. Post-induction, cells were pelleted at 10,000 × *g* and the supernatant was decanted. Pellets were resuspended in 5 mL of lysis buffer (20 mM Tris, 300 mM NaCl, 10% glycerol, 50 µM ZnSO_4_) and lysed mechanically with glass beads in a FastPrep-24 instrument. Recombinant proteins were then purified by immobilized metal affinity chromatography (His-Select Nickel Affinity Gel, Sigma-Aldrich) and eluted with a 100 mM imidazole lysis buffer. Imidazole was removed using a Vivaspin 3000 MWCO (Sigma-Aldrich) and AdcR-His was concentrated in a dialysis buffer (20 mM Tris, 50 mM NaCl, 10% glycerol, 50 µM ZnSO_4_).

### Electrophoretic mobility shift assay

Promoter region of *sak_RS01240* and *rpsNb* were amplified by PCR using the primers listed in the Table S5. The *murG* promoter was used as a negative control. The EMSA mix was prepared in a final volume of 15 µL with increasing concentrations of AdcR-His proteins and 20 ng of DNA. The binding buffer was prepared following the indications of reference 50. Samples were incubated for 30 minutes at 25°C, loaded onto a 10% acrylamide gel, and run for 4 hours at 100 V in 0.5% TAE buffer. Gels were stained in a 0,.025% BET solution.

### Preparation of protein extracts and Western blot analyses

Bacteria were grown in 10 mL of CDM medium and harvested (10 mL samples) during the mid-exponential phase of growth (OD_600_ = 0.4). Cells were resuspended in 500 µL of Z buffer (49) and lysed mechanically with glass beads using FastPrep-24 instruments. Cell debris was eliminated by centrifugation (5 min, 6,000 rpm). Protein concentrations were determined using the Bio-Rad protein assay (Bio-Rad, Hercules, CA). For each sample, the same amount of total cell protein extract was loaded and separated on SDS-polyacrylamide 12% gel electrophoresis gels and electrotransferred on the Hybond-C extra transfer membrane (Amersham). Coomassie Brilliant Blue staining was done in parallel as a protein loading control. 3×Flag was detected using corresponding monoclonal anti-Flag M2 antibody, dilution 1:2,000 (Sigma). Immunodetection was carried out with anti-mouse immunoglobulin G-peroxidase antibody, dilution 1:1,000 (Sigma), followed by visualization (at 540 nm) using a Fluorimager 595 (Amersham Biosciences).

### Competition assays in human biological fluids

To easily discriminate the wild-type and mutant strains in mixed cultures, we used the pTCV-*lac* vector, which carries a promoterless *lacZ* gene, or the pTCV-lac-Pcyl vector with a lacZ gene under the control of the strong and constitutively active promoter Pcyl (51). When plated on TH agar containing erythromycin (10 µg mL^−1^) and X-Gal (60 µg mL^−1^), strains carrying pTCV-*lac*-P*cyl* vector appeared as blue colonies while strains carrying pTCV-*lac* remained white.

Each strain was transformed with pTCV-*lac* and pTCV-*lac*-P*cyl* plasmids. Mixed cultures were inoculated alternately with wild-type strain carrying pTCV-*lac*/mutant strain carrying pTCV-*lac*-P*cyl* combination or wild-type-carrying pTCV-*lac*-P*cyl*/mutant strain carrying pTCV-*lac* to ensure that plasmids had no deleterious effect on bacterial growth. All strains were grown during 8 hours in TH with erythromycin 10 µg mL^−1^, then in zinc-restricted CDM with erythromycin 10 µg mL^−1^ overnight, and mixed cultures were inoculated with approximately 5 × 10^6^ CFU mL^−1^ of each strain in human biological fluids supplemented with erythromycin 10 µg mL^−1^. Bacterial growth was monitored during 24 hours and wild-type and mutant strains were discriminated by plating culture dilutions on TH agar containing erythromycin (10 µg mL^−1^) and X-Gal (60 µg mL^−1^) using the EddyJet2 spiral plater (I&L Biosystems). For each competition experiment between the WT and a mutant strain, both combinations (WT pTCV-*lac*/Δ pTCV-*lac*-P*cyl* or WT pTCV-*lac*-P*cyl*/Δ pTCV-lac) were tested. Each experiment was repeated at least three times.

Human serum was obtained from healthy donors of the Etablissement Français du Sang (EFS centre Atlantique, France). It was decomplemented by heating at 56°C for 30 min. Amniotic fluid and cerebrospinal fluid (CSF) were collected in the Bretonneau University Hospital (Tours, France) from, respectively, 2 and 3 donors who were not taking medications that would influence the analysis, such as antimicrobial agents.

## SUPPLEMENTAL MATERIAL

Supplemental table captions msphere.00614-24-s0001.docx Captions for Tables S1 to S3.

Supplemental material msphere.00614-24-s0002.pdf Tables S4 and S5 and Fig. S1 to S7.

Table S1 msphere.00614-24-s0003.xlsx Transcriptomic analyses, 0 versus 10 µM Zn.

Table S2 msphere.00614-24-s0004.xlsx Transcriptomic analyses, 0 versus 300 µM Zn.

Table S3 msphere.00614-24-s0005.xlsx Transcriptomic analyses, 300 versus 10 µM Zn.

Table S6 msphere.00614-24-s0006.pdf Location and sequence of AdcR boxes located within promoter region in *S. agalactiae*.

ASM does not own the copyrights to Supplemental Material that may be linked to, or accessed through, an article. The authors have granted ASM a non-exclusive, world-wide license to publish the Supplemental Material files. Please contact the corresponding author directly for reuse.
