# Supplementary material for: Adaptation to zinc restriction in Streptococcus agalactiae: role of the ribosomal protein and zinc-importers regulated by AdcR
Source: mSphere. 2024 Oct 31;9(11):e00614-24. doi: 10.1128/msphere.00614-24 (PMC11580457; doi:10.1128/msphere.00614-24)
Supplement: Supplemental table captions — Captions for Tables S1 to S3. [file msphere.00614-24-s0001.docx]

**Supplemental material legends**

**Table S1 Genes differentially expressed in *S. agalactiae* grown in Zn-restricted conditions (0 µM Zn), as compared to Zn-replete conditions (10 µM ZnSO_4_)**

**Table S2 Genes differentially expressed in *S. agalactiae* grown in Zn-replete conditions (10 µM Zn), as compared to Zn-excess conditions (300 µM ZnSO_4_)**

**Table S3 Genes differentially expressed in *S. agalactiae* grown in Zn-restricted conditions (0 µM Zn), as compared to Zn-excess conditions (300 µM ZnSO_4_)**

Mapped read counts were generated using HTSeq-count (46) and differentially expressed genes were defined using DESeq2 (47). Genes with an adjusted P-value of ≤0.05 and fold change (FC) ≥2 were considered significantly modulated and are highlighted in color.
