# Supplementary material for: Adaptation to zinc restriction in Streptococcus agalactiae: role of the ribosomal protein and zinc-importers regulated by AdcR
Source: mSphere. 2024 Oct 31;9(11):e00614-24. doi: 10.1128/msphere.00614-24 (PMC11580457; doi:10.1128/msphere.00614-24)
Supplement: Supplemental material — Tables S4 and S5 and Fig. S1 to S7. [file msphere.00614-24-s0002.pdf]

Table S4 Strains and plasmids

Strain or plasmid	Genotype or description	Source or reference
Escherichia coli		
XL1-blue	*endA1 gyrA96* (Nal^{R}) *thi-1 recA1 relA1 lac glnV44 hsdR17*(r$_{K}^{-}$ m$_{K}^{+}$) *F'* [::Tn10 (Tet^{R}) *proAB*$^{+}$ lacI^{q} ZΔM15]	Stratagene
BL21 codon + (DE3)-RIL	*F- dcm ompT hsdS (r B - m B -) gal [malB+] K-12 (λS)*	Novagen
Streptococcus agalactiae		
A909	Isolated from a septic human neonate in 1934	(Tettelin *et al.*, 2005)
A909Δ*adcR*	Isogenic *adcR (sak_RS01075)* deletion mutant	(Moulin et al., 2016)
A909Δ*adc*/*lmb*	Isogenic *lmb*, *adcA, adcAII* and *adcCB* deletion quintuple mutant	(Moulin et al., 2016)
A909Δ*rpsNb*	Isogenic *rpsNb* (*sak_RS08945*) deletion mutant	This study
A909Δ*rpsNb*^{c}	Chromosomic complementation of the Δ*rpsNb* strain	This study
A909Δ *sak_RS00910*	Isogenic *sak_RS00910* deletion mutant	This study
A909Δ*sak_RS01240-RS01260*	Isogenic *sak_RS01240-RS01260* deletion mutant	This study
A909Δ*adc*/*lmb*Δ *sak_RS01240-RS01260*	Isogenic *lmb*, *adcA*, *adcAII*, *adcCB* and *sak_RS01240-RS01260* deletion mutant	This study
<u>Plasmids</u>		
pGhost1^{TS}	Replication-thermosensitive shuttle (^{TS}) plasmid, Ery^{R}	(Biwas *et al.,* 1993)
pTVC-P$_{tet}$	Mob+(IncP);oriR pACYC184;oriR pAM_1; complementation vector, promoter P*tet*	(Firon *et al.,* 2013)
pTVC-P$_{tet}$ *rpsNb*	*rpsNb* complementation vector, promoter P*tet*	This study
pTCV-*lac*	Promoter probe plasmid carrying a *lacZ* gene devoided of a promoter and the *ermB* gene (Ery^{R})	(Poyart and Trieu-Cuot, 1997)
pTCV-*lac*-P$_{cyl}$	Plasmid carrying a *lacZ* gene under the control of the constitutive promoter Pcyl	(Firon *et al.,* 2013)
pTCV-*lacZ*::P*rpsNb*	pTCV*lacZ* containing the promoter region of *rpsNb* upstream of the *lacZ* gene	This study
pTCV-*lacZ*:: P*rpsNbmutbox1**	pTCV-*lacZ*::P*rpsNb* with 5 nucleotide substitutions in the AdcR box1.	This study
pTCV-*lacZ*:: P*rpsNbmutbox2**	pTCV-*lacZ*::P*rpsNb* with 5 nucleotide substitutions in the AdcR box2.	This study
pTCV-*lacZ*:: P*rpsNbmutbox3**	pTCV-lacZ::P*rpsNb* with 5 nucleotide substitutions in the AdcR box3.	This study
pTCV-*lacZ*:: P*rpsNbmutbox2-3**	pTCV-lacZ::P*rpsNb* with nucleotide substitutions in the AdcR box2 and 3.	This study

pTCV-lacZ:: P*rpsNbmutbox1-2-3**	pTCV-lacZ::P*rpsNb* with nucleotide substitutions in the AdcR box1-2 and 3.	This study
pTCV-*lacZ*:: P*rpsNbmutbox2-3**with T_4::A substitution in the box1	pTCV-lacZ::P*rpsNb* with nucleotide substitutions in the AdcR box2 and 3 and a T4::A substitution in the box1.	This study
pTCV-lacZ:: P*rpsNbmutbox2-3** with T_4::A and G_8::A substitution in the box1	pTCV-lacZ::P*rpsNb* with nucleotide substitutions in the AdcR box2 and 3 and a T_4::A and G_8::A substitutions in the box1.	This study
pTCV-lacZ:: P*rpsNb*mutbox2-3*with T_4::G substitution in the box1	pTCV-lacZ::*rpsNb* with nucleotide substitutions in the AdcR box2 and 3 and a T_4::G substitution in the box1.	This study
pTCV-*lacZ*::P*sak_RS01240*	pTCV-*lacZ* containing the promoter region of *sak_RS01240* upstream of the *lacZ* gene	This study
pTCV-l*acZ*::P *sak_RS01240mutbox*	pTCV-*lacZ*::P*sak_RS01240* with 5 nucleotide substitutions in the AdcR box	This study
pTCV-*lacZ*::P*adhP*	pTCV-lacZ containing the entire promoter region of *adhP* upstream of the *lacZ* gene	This study
pTCV-lacZ::P*adhPmutbox*	pTCV-*lacZ*::P*adhP* with 5 nucleotide substitutions in the AdcR box	This study
pTCV-lacZ::RpsNa-3XFlag	pTCV-lacZ containing the promoter and the *rpsNa* coding region fused to 3XFlag	This study
pTCV-lacZ::RpsNb-3XFlag	pTCV-lacZ containing the promoter and the *rpsNb* coding region fused to 3XFlag	This study
pET28a ::*adcR*	pET28a containing the *adcR* gene	This study

Table S5 Primers

Added restriction site sequences are shown in bold
Nucleotides targeted for site-directed mutation are shown in red

Primer	Description	5' →3' sequence
**Primers for deletion mutants**		
OAH365	*rpsNb* (*sak_RS08945*) upstream and downstream region	GATACT**GGATCC**GTGAACTCTGTGAAATCAAG
OAH366		**TGGTCTCG**ATTTCTCCTTTCCCAAAAA
OAH367		**TGGTCTCG**AAATGGTAATTCGTGAAGACAAA
OAH368		GTTTAC**GAATTC**TGTAAAGCAGCATGGTG
OAH432	*sak_RS00910* upstream and downstream region	TAT**GGATCC**TGCTTTGCGTTCATCAGTTCCTAA
OAH433		AAA**GCATGC**CTTTTTTCCGTCTCTCCAATTATAA
OAH434		AAA**GCATGC**GACAATTTATTAACGTTAACAGACACCA
OAH435		TGA**GGTACC**GTTTCTGGACGTTCTTCAATGGA
OAH375	*sak_RS01240-RS01260* upstream and downstream region	TATTTC**CTGCAG**GGTTGGGCTTAGTCAAGTCGG
OAH376		T**GGTCTC**GCAAATATTTAGTCACTATTAAACCTCC
OAH377		T**GGTCTC**GTTTGTGGAGATTAAAAAACTCACCTACT
OAH378		TGTAAA**CCCGGG**AGCCAGCATTGTCAACACACGC
**Primers for complementation**		
OAH414	*rpsNb* coding region	AAT**GGATCC**GGGAAAGGAGAAATCATGGCTAAG
OAH415		TTT**CTGCAG**AAAAACTTTGAGCAATTTGCTCAAAGTTTTTGAAT TACCAGCTTGCTTTTTTAATGC
**Primers for *lacZ*-transcriptional fusions**		
OAH392	P*rpsNb* Promoter region	CTT**GGATCC**GATTTCTCCTTTCCCAAAAAGAT
OAH413		TTGTA**GAATTC**AAAAACTTTGAGCAATTTGCTCAAAGTTTTTTC A TCACCATTACAATATAATACTTATCC
OAH407	P*rpsNb* mutbox1*	TTGTA**GAATTC**AAAAACTTTGAGCAATTTGCTCAAAGTTTTTTC A TCACCATTACAATATAATACCCCCTCAGTT
OAH408	P*rpsNb* mutbox2*	**TGGTCTCGGGGG**CAAGACATGATTCTATTATACTCTTTTC
OAH409		**TGGTCTCG**CCCCTTGGTTAATTACCAGTTAACTAGGCA
OAH410	P*rpsNb* mutbox3*	**TGGTCTCAGGGT**TGTTAATTAACCAGTAAACAAGACAT
OAH411		**TGGTCTCT**ACCCCCTAGGCACCCTGTTTTGG
OAH542	P*rpsNb* box1 with T$_4$::A substitution	TTGTA**GAATTC**AAAAACTTTGAGCAATTTGCTCAAAGTTTTTTC A TCACCATTACAATATAATACTTAACCAGTT
OAH594	P*rpsNb* mutbox1 with T$_4$::A and G$_8$::A substitution	TTGTA**GAATTC**AAAAACTTTGAGCAATTTGCTCAAAGTTTTTTC ATCACCATTACAATATAATACTTAACCAATT
OAH595	P*rpsNb* mutbox1 with T$_4$::G substitution	TTGTA**GAATTC**AAAAACTTTGAGCAATTTGCTCAAAGTTTTTTC ATCACCATTACAATATAATACTTAGCCAGTT
OAH506	P*sak_RS01240* Promoter region	GGTTTG**GAATTC**CTAATAGCGTGGTTAAGACTG
OAH507		ATATTT**GGATCC**TATTAAACCTCCTTTTTTAATTGATAA
OAH538	P*sak_RS01240* mutbox*	**TGGTCTCG**TTAGAAAATTTGTTATTTTATCATTAATAAC
OAH539		**TGGTCTCGCTAA**CCCCTCAGTTAAGTAATTGTTCTCCTACTT
OAH534	P*adh*	TGAT**GGATCC**AAAACCTCCTAGAAATAAATA

OAH543	(*sak_RS00430*) Promoter region	ATTC**GAATTC**AAAAAGATTAACCAGTTAAGAATATTT
OAH536	*Padh* mutbox	TGAT**GGATCC**AAAACCTCCTAGAAATAAATA
OAH544		ATTC**GAATTC**AAAAAGACCCCTCAGTTAAGAATATTT
OAH552	p*SAK_RS09650*	AATA**GAATTC**CAATTATACATGATAGACACGAC
OAH553		ACGT**GGATCC**TGTGTAATTCCTTTCTTCGTTATC
OAH554	p*SAK_RS05875*	AAAA**GAATTC**ACGCCATCCCTTTTTAGTCATT
OAH555		CAAA**GGATCC**TATTTTCTTGGGTATTAGCATATGAAC
OAH556	p*SAK_RS05270*	TTTT**GAATTC**CATTAAAAAATGCCCCTTTTCTTC
OAH557		TGCA**GGATCC**TCTAAACATCTCCTTAAATATTTTTGGG
OAH558	*PadcR* Promoter region	TTGC**GAATTC**GACAGGAAGTGGACCAACT
OAH559		AAAC**GGATCC**ATATACCTCTTTTTTGTTAACCAGT
OAH581	p*SAK_RS09200*	GAA**GAATTC**TTATAAATGAGTTAACCGTTTAACTTAA
OAH582		TCT**GGATCC**ATCCTCCTTTGGTGAAG
OAH585	p*SAK_RS04420*	ACC**GAATTC**ATCACTTCCTCTCTTAA
OAH586		GCG**GGATCC**ATACTCCTTTAATAATA
OAH584	p*SAK_RS00915*	GTT**GGATCC**CATCTGCTTTCTATACT
OAH589		AAA**CCCGGG**GACAATTTATTAACG TTAACAGACACCA
OAH590	p*SAK_RS04000*	CTTT**GAATTC**TATGGTTAGAGTTGATATGATAAACTG
OAH591		TTC**GGATCC**AAAAGAACCTCACATTACTTAACC
OAH592	p*SAK_RS04070*	TTTT**GAATTC**CAAGCGTCTACCATATTGATATCC
OAH593		TTT **GGATCC**AGAACAAGCCTCCTTACCACTATC
**Primers for protein purification**		
OAH496	AdcR-his	TTT**CCATGG**TTACAGTTTTAGAACAAAAATTAGACCA
OAH497		CGCA**CTCGAG**CCCTTCTAATTCTCTAGAGAAAAG
**Primers for 3XFlag protein fusion**		
OAH567	RpsNa-3XFlag	TCAT**GAATTC**ATTCAAAAAATGTAACCAGAAAT
OAH569		**TGGTCTCA**ATTTACGATAATAGGCTAGCTCC
OAH570		**TGGTCTC**AAAATTCTAGGAGGTAAATAAATTGGCT
OAH571		**TGGTCTC**AAGTCCCAAGATGCTTTAGTTACGCCTG
OAH572		**TGGTCTC**AGTAATAAATTAATGATGCTTTGTGTTTAAAAG
OAH573		TGCCTTGTTTGTCATCTTC
OAH574		TCAT**GGATCC**TTTTTAATTCTCCTCTTATTAGCAG
OAH391	RpsNb-3XFlag	TTGTA**GAATTC**TCATCACCATTACAATATAATACTTATCC
OAH577		**TGGTCTC**AAGTCCCAGCTTGCTTTTTTAATGCC
OAH578		**TGGTCTCA**GTAATTCGTGAAGACAAATAGTTACTTT
OAH579		AAAA**GGATCC**TATCTTGGCATACCGGGC
**Primers for qRT-PCR**		
OAH001	*adcR*	GACTAATACGCAAGAACACATTCT
OAH008		CTGCTTTAGTTACAGCTGCCTG

OAH051	*adcAII*	CTATTGCAGAGAAGTATAAGCCAAAA
OAH052		GCTAAGTATGAGAATGCTGTATGTGAAG
OAH355	*rpsN*	TGCGTAAATTCGGTATGAGTCG
OAH356		TGGCCTTTGTAAGCTAGGTT
OAH351	*sak_RS01240*	CGAAATCCCTATTGGCACGG
OAH352		CTCTGGCGTTGCGTAAATCA
OAH361	*adhP*	TTTGGGTCACGAAGGCATTG
OAH362		CAGTATTCGCAATGACCGCA
OLM317	*16S*	GCAACGCGAAGAACCTTACC
OLM318		CTCTAGGCCGGTCAGAAGGAT

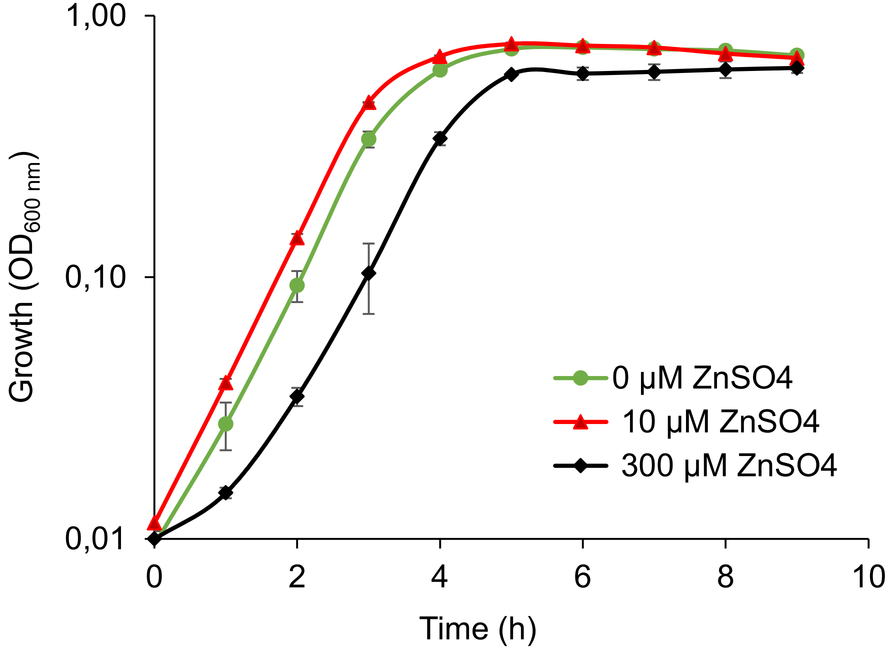

Figure S1: **Growth of wild-type *S. agalactiae* A909 in CDM supplemented with 0, 10 or 300 µM $ZnSO_4$** .

Growth was monitored by OD_{600nm} measurements every 60 min for 10 h. The data are presented as mean OD_{600nm} measurements ± the standard deviations from three independent experiments.

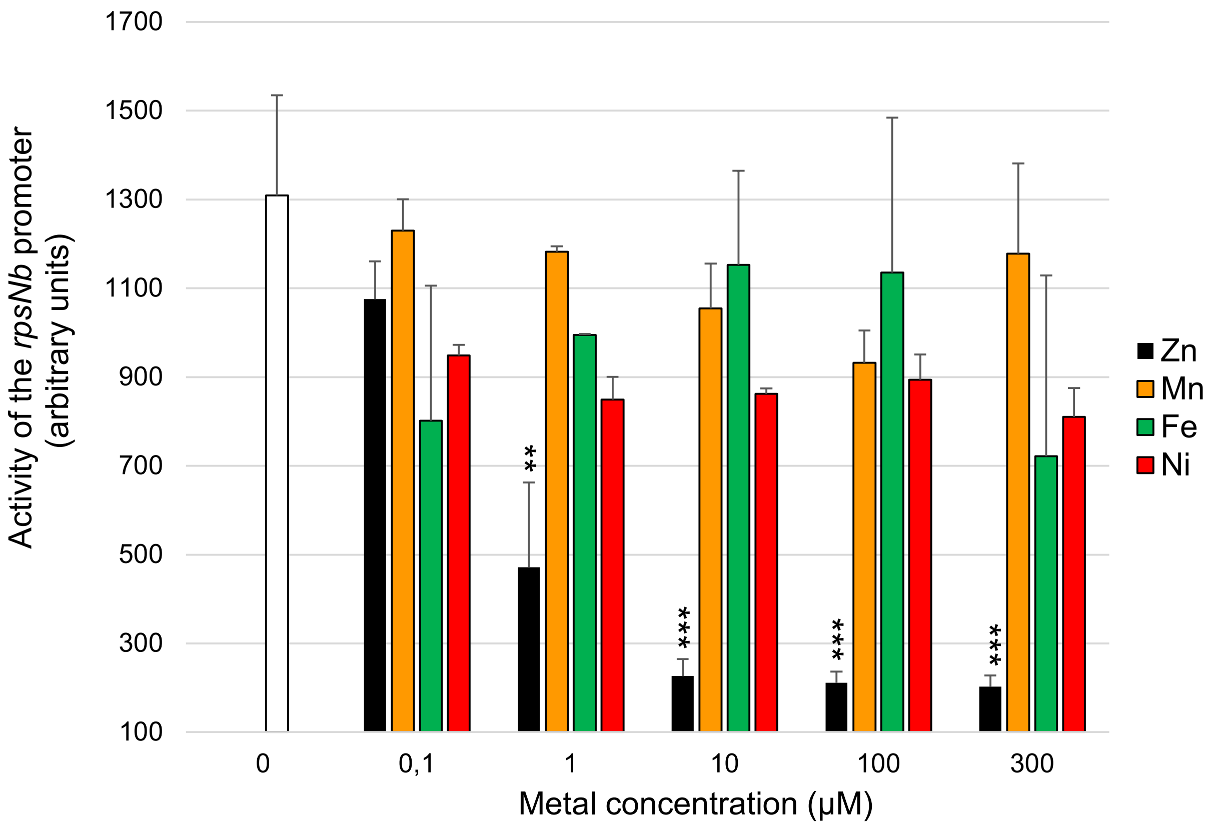

Figure S2: Expression of the *S. agalactiae rpsNb* gene is Zn dependent.

The *rpsNb* promoter activity was measured in Zn-restricted CDM supplemented with various amounts of added metals (0 to 300 µM). Cells containing the P_{rpsNb}-*lacZ* transcriptional fusions were grown until the mid-exponential phase of growth (OD_{600}, 0.5), and β-galactosidase assays performed. The values shown are mean results ± standard deviations. The asterisks indicate *P* values obtained using unpaired Student's *t* test, comparing promoter activity of cells grown in zinc-restricted CDM and cells grown in CDM with the various added $ZnSO_4$ concentrations. **, $P < 0.01$; ***, $P < 0.001$.

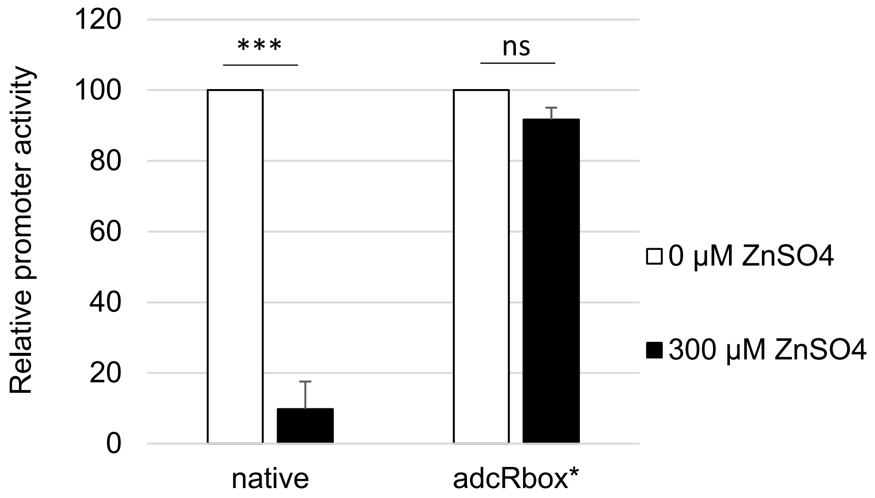

Figure S3: The *sak_RS01240* promoter is repressed in the presence of zinc through an AdcR binding site

Transcriptional *lacZ* fusions with either the native *sak_RS01240* promoter region or the *sak_RS01240* promoter region containing point mutations destroying its AdcR-binding site (*adcRbox**) were constructed and introduced into the WT strain. β-Galactosidase assays were performed from bacteria grown in Zn-restricted CDM (white bars) or in CDM containing 300 µM of added $ZnSO_4$ (black bars). Results are expressed as relative activity. Promoter activity of bacteria grown in Zn-restricted CDM (white bars) was used as reference (100%) . The values shown are the means ± standard deviations of three independent assays. The asterisks indicate P values obtained using an unpaired Student t test compared to the promoter activity of bacteria grown in Zn-restricted conditions. ***, $P < 0.001$; ns, non significant.

A.

```
RpsNb  MAKKSKIAKFQKQQKLVEQYAELRRELKEKGDYEALRKLPKDSNPNRLKNRDLIDGRPHAYMRKFGMSRINFRNLAYKGQIPGIKKASW
RpsNa  MAKKSMIAKNKRPAKFSTQAYTRCEKCG----------------------------RPHSVYRKFQLCRVCFRDLAYKGQVPGVTKASW
       ***** *** ::  *:  *     .:                              ***:  *** :.*: **:******:**:.****
```

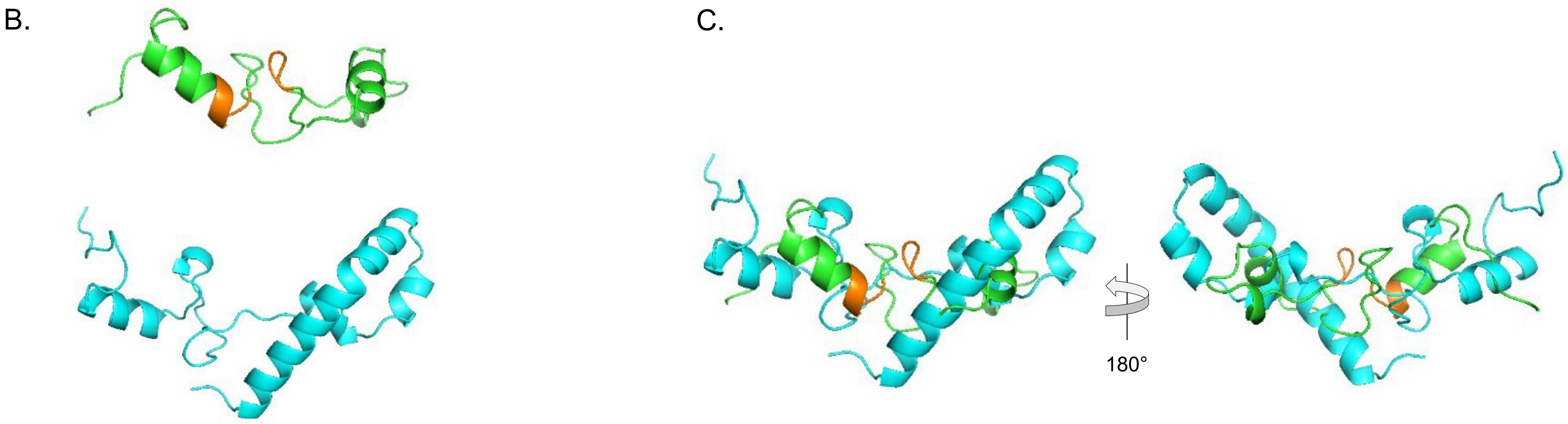

Figure S4: Comparison between RpsNa and RpsNb proteins

A. Alignment between *S. agalactiae* RpsNa and RpsNb proteins

* denotes identical amino acid residues. The positions of the Zn-ribbon motif are boxed in red. The alignment was performed using the CLUSTLAW program.

B. Structural modeling of RpsNa in green and RpsNb in blue and their merge (C) . Zn binding sites of RpsNa are labelled in orange. Models were realized using the PyMOL software.

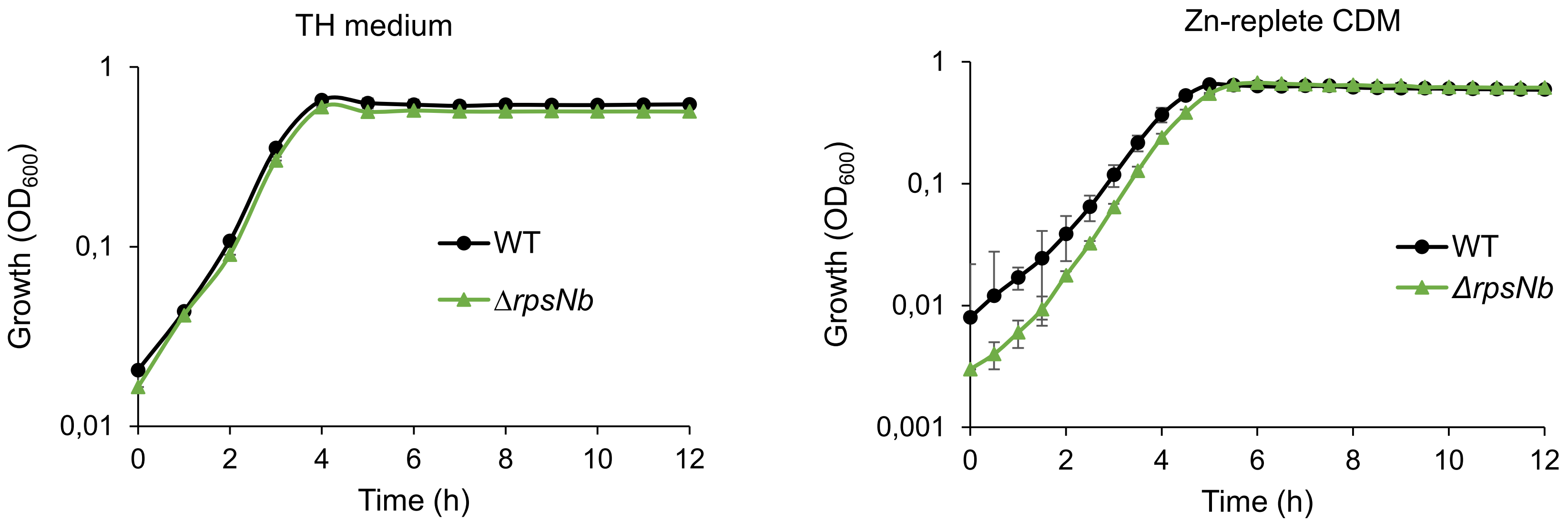

Figure S5: Growth of *S. agalactiae* A909 WT and Δ*rpsNb* mutant in TH medium and in Zn-replete CDM (10 µM $ZnSO_4$).

Growth was monitored by OD_{600nm} measurements every 60 min for 12 h. The data are presented as mean OD_{600nm} measurements ± the standard deviations from three independent experiments.

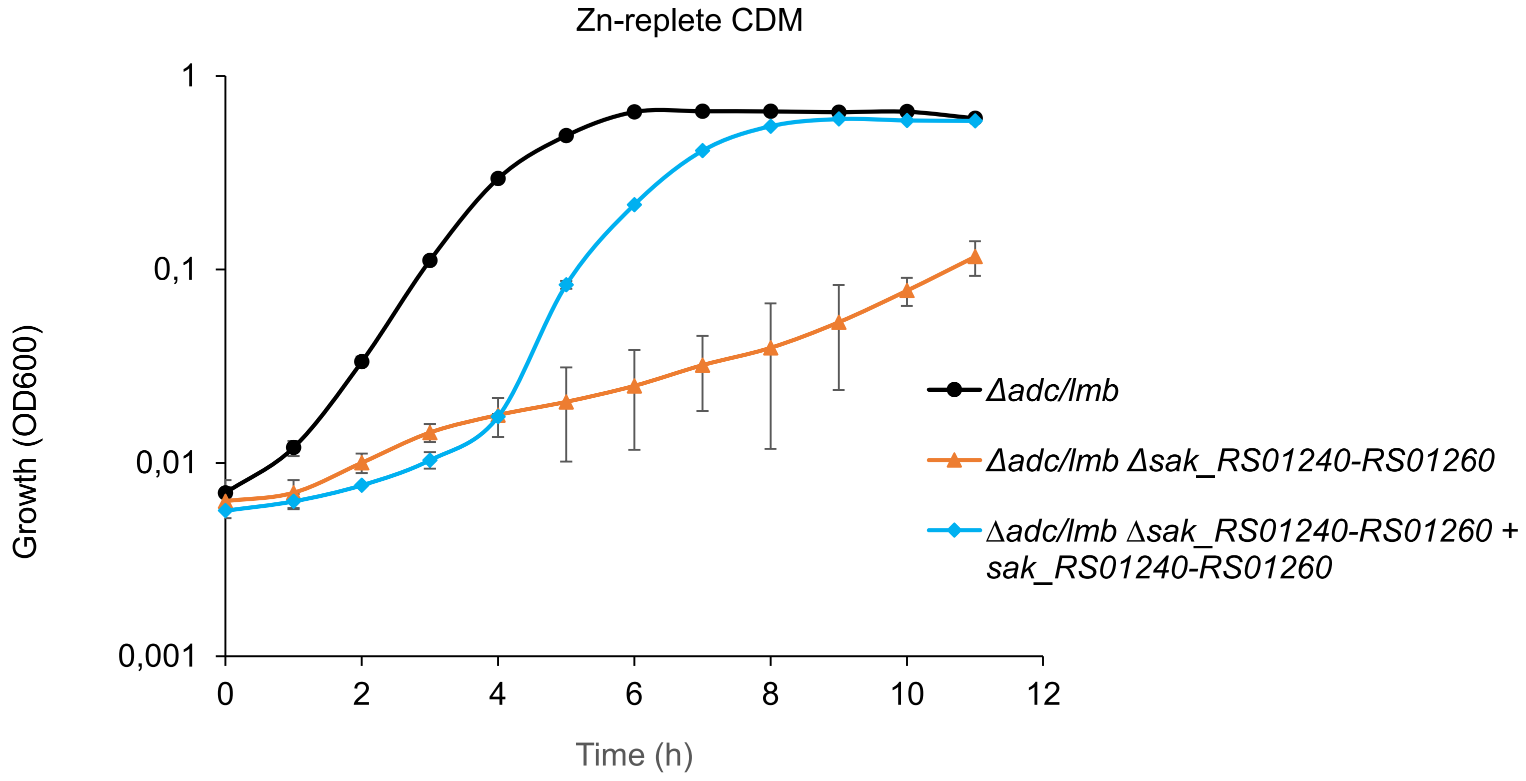

Figure S6: Growth of the *sak_RS01240-RS01260* complemented strain in Zn-replete CDM (10 μM $ZnSO_4$).

Growth was monitored by OD_{600nm} measurements every 60 min for 12 h. The data are presented as mean OD_{600nm} measurements ± the standard deviations from three independent experiments.

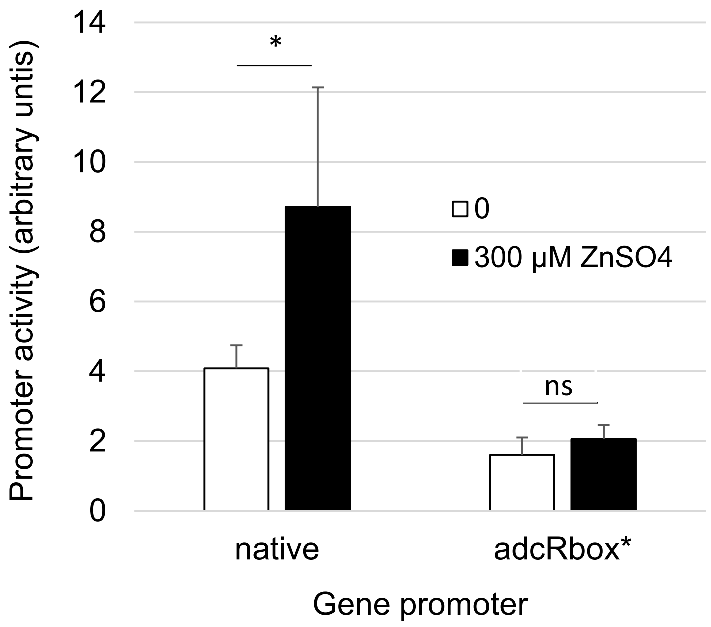

Figure S7: The *adhP* promoter is induced in the presence of Zn through an AdcR binding site.

Transcriptional *lacZ* fusions with either the native *adhP* promoter region or the *adhP* promoter region containing point mutations destroying its AdcR-binding site (AdcR-box*) were constructed and introduced into the WT strain. β-Galactosidase assays were performed as described in Materials and Methods. The relative activity of the promoters was measured either in zinc-restricted CDM (white bars) or in CDM containing 300 µM of added $ZnSO_4$ (black bars). The values shown are the means ± standard deviations of three independent assays. The asterisks indicate *P* values obtained using an unpaired Student *t* test compared to the promoter activity of bacteria grown in zinc restricted conditions. *, $P < 0.05$; ns, non significant.
