# Supplementary material for: Adaptation to zinc restriction in Streptococcus agalactiae: role of the ribosomal protein and zinc-importers regulated by AdcR
Source: mSphere. 2024 Oct 31;9(11):e00614-24. doi: 10.1128/msphere.00614-24 (PMC11580457; doi:10.1128/msphere.00614-24)
Supplement: Table S6 — Location and sequence of AdcR boxes located within promoter region in S. agalactiae. [file msphere.00614-24-s0006.pdf]

## Table S6: Location and sequence of AdcR boxes located within promoter region in *S. agalactiae*

| Gene | Product | Position of AdcR box[1]<br>*Star indicate mismatch with the consensus sequence TTAACNNGTTAA Only sequences containing mismatch are indicated* | Significant fold change in RNA seq | Zn and AdcR-regulation validated[2] |
|---|---|---|---|---|
| **Zinc-repressed genes** | | | | |
| *sak_RS00910* | Hypothetical protein | TTTACCAGTTAA<br>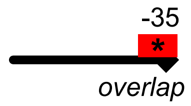 | Yes<br>(0 vs 10 µM<br>0 vs 300 µM) | Yes |
| *rpsNb*<br>*sak_RS08945* | 30S ribosomal protein S14 | TTATCCAGTTAA<br>TTTACTGGTTAA<br>ATTACCAGTTAA<br>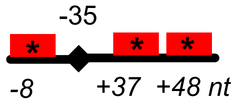 | Yes<br>(0 vs 10 µM<br>0 vs 300 µM) | Yes |
| *sak_RS08940*<br>(Antisense of *rpsNb*) | DUF4931 containing protein | 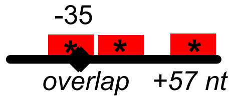 | Yes<br>(0 vs 300 µM) | Yes |
| *adcAII*<br>*sak_RS09555*<br>*shtII*<br>*sak_RS09550* | Zn-transporter solute-binding histidine triad protein | 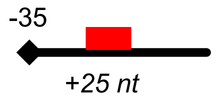 | Yes<br>(0 vs 10 µM<br>0 vs 300 µM) | Yes |
| *lmb*<br>*sak_RS06625*<br>*sht*<br>*sak_RS06620* | Zn-transporter solute-binding histidine triad protein | ATAACTGGTTAA<br>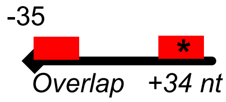 | No | Yes |
| *adcA*<br>*sak_RS03425* | Zn -transporter substrate-binding | TTAACCGGTAAA<br>ATAACGGGTTAA<br>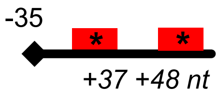 | Yes<br>(0 vs 300 µM) | Yes<br>(Moulin *et al.* 2016) |
| *adcR*<br>*sak_RS01075*<br>*adcCB*<br>*sak_RS01080*<br>*sak_RS01085* | MarR family regulator<br>Zn transporter ATP-binding permease | TTTACTGGTTAA<br>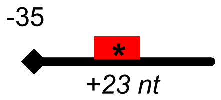 | Yes<br>(0 vs 300 µM) | Yes |
| *sak_RS01240* | ABC transporter - binding protein | 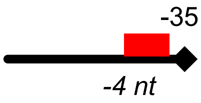 | Yes<br>(0 vs 10 µM<br>0 vs 300 µM) | Yes |

| | | | | |
|---|---|---|---|---|
| *sak_RS07770* | ABC transporter substrate binding protein | -35 *overlap* | Yes (0 vs 300 µM) | Yes |
| *sak_RS07375* | Ammonium transporter | -35 *-4 nt* | Yes (0 vs 10 µM 0 vs 300 µM) | ND |
| *sak_RS07380* | Flavoprotein involved in K+ transport | -35 *+9 nt* | Yes (0 vs 10 µM 0 vs 300 µM) | ND |
| *sak_RS07385 antisense of sak_RS07380* | Rhodanese-related sulfur-transferase | -35 *overlap* | Yes (0 vs 10 µM 0 vs 300 µM) | ND |
| *sak_RS01090* | Putative DNA/RNA endonuclease | TTAACTAGTTAT -35 * *-5 nt* | Yes (0 vs 10 µM 0 vs 300 µM) | Yes |
| *sak_RS04480* | LPXTG cell wall anchor domain-containing protein | -35 *+66 nt* | Yes (0 vs 300 µM) | ND |
| *sak_RS05125* | Pneumococcal-type histidine triad protein | -35 *+22 nt* | No | Yes |
| *sak_RS04000* | Hypothetical protein | TTAGCTGGTTAA -35 * *+4 nt* | No | Yes |
| **Zinc-induced genes** | | | | |
| *adhP sak_RS00430* | Alcohol de-hydrogenase | -35 *-31 nt* | Yes (0 vs 300 µM) | Yes |
| **No zinc regulation observed** | | | | |
| *sak_RS04070* | FAD/NAD(P)-binding protein | TTTACTCGTTAA -35 * *-37 nt* | Yes (0 vs 300 µM) | No |
| *sak_RS04075* | DNA/RNA non-specific endonuclease | TTTACTCGTTAA -35 * *-46 nt* | No | ND |

| | | | | |
|---|---|---|---|---|
| *sak_RS10550* | Site-specific integrase | TTAACCAGGTAA<br>-35<br>*<br>*+133 nt* | No | ND |
| *sak_RS00915*<br>antisense of<br>*sak_RS00910* | IS30-like element ISSag3 family transposase | TTTACCAGTTAA<br>-35<br>*<br>*-17 nt* | Yes<br>(0 vs 300 µM) | No |
| *pfkA*<br>*sak_RS05185* | 6-phospho-fructokinase | TTAACGATTTAA<br>-35<br>*<br>*+23 nt* | Yes<br>(0 vs 300 µM) | No |
| *sak_RS09200*<br><br>*sak_RS09205* | Hypothetical protein<br>LacI family regulator | TTAAACGGTTAA<br>TTAACCGTTTAA<br>-35<br>* *<br>*-58 +16 nt* | No | No expression detected |
| *pepF*<br>*sak_RS04420* | Oligoendopeptidase F | TTAACTGATTAA<br>-35<br>*<br>*+37 nt* | No | No |
| *ppc*<br>*sak_RS04425* | Phosphoenol-Pyruvate carboxylase | TTAACTGATTAA<br>-35<br>*<br>*-103 nt* | No | ND |
| *sak_RS06655* | AbiH family protein | TAAACCTGTTAA<br>-35<br>*<br>*-23 nt* | No | ND |
| *sak_RS06425* | AI-2E family transporter | TTAACACGTTAT<br>-35<br>*<br>*+11 nt* | No | No expression detected |
| *mvk*<br>*sak_RS06830* | Mevalonate kinase | TTATCACGTTAA<br>-35<br>*<br>*overlap* | No | No |

| | | | | |
|---|---|---|---|---|
| *sak_RS01585* | Hypothetical protein | TTTACATGTTAA 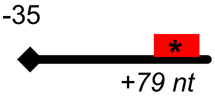  | No | ND |
| *sak_RS05655* | Hypothetical protein | TTAACTCCTTAA 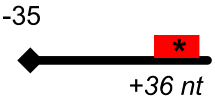  | No | ND |

1. Bioinformatics analysis was performed on the genome of the A909 *S. agalactiae* strain using MEME software (https://meme-suite.org/meme/doc/meme.html). AdcR putative binding sites (5′-TTAACNNGTTAA-3′;1 mismatch allowed) were searched within intergenic regions (only AdcR box located at less than 200 nt of the -35 promoter element were included).
2. ND: Not determined
